# Rapid classification of group B Streptococcus serotypes based on matrix-assisted laser desorption ionization-time of flight mass spectrometry and machine learning techniques

**DOI:** 10.1186/s12859-019-3282-7

**Published:** 2019-12-24

**Authors:** Hsin-Yao Wang, Wen-Chi Li, Kai-Yao Huang, Chia-Ru Chung, Jorng-Tzong Horng, Jen-Fu Hsu, Jang-Jih Lu, Tzong-Yi Lee

**Affiliations:** 1 Department of Laboratory Medicine, Chang Gung Memorial Hospital at Linkou, Taoyuan, 33305 Taiwan; 2 Program in Biomedical Engineering, Chang Gung University, Taoyuan City, Taiwan; 3 Warshel Institute for Computational Biology, The Chinese University of Hong Kong, Shenzhen, 518172 China; 4 Department of Computer Science and Information Engineering, National Central University, Taoyuan, 32001 Taiwan; 5 Department of Bioinformatics and Medical Engineering, Asia University, Taoyuan City, Taiwan; 6 Division of Pediatric Neonatology, Department of Pediatrics, Chang Gung Memorial Hospital, Linkou, Taoyuan, 33305 Taiwan; 7 School of Traditional Chinese Medicine, College of Medicine, Chang Gung University, Taoyuan, 33302 Taiwan; 8 Department of Medical Biotechnology and Laboratory Science, Chang Gung University, Taoyuan, Taiwan; 9 Department of Medicine, College of Medicine, Chang Gung University, Taoyuan, Taiwan; 10 School of Life and Health Sciences, The Chinese University of Hong Kong, Shenzhen, 518172 China

**Keywords:** Group B streptococcus, GBS, Serotypes, MALDI-TOF-MS, Machine learning

## Abstract

**Background:**

Group B streptococcus (GBS) is an important pathogen that is responsible for invasive infections, including sepsis and meningitis. GBS serotyping is an essential means for the investigation of possible infection outbreaks and can identify possible sources of infection. Although it is possible to determine GBS serotypes by either immuno-serotyping or geno-serotyping, both traditional methods are time-consuming and labor-intensive. In recent years, the matrix-assisted laser desorption ionization-time of flight mass spectrometry (MALDI-TOF MS) has been reported as an effective tool for the determination of GBS serotypes in a more rapid and accurate manner. Thus, this work aims to investigate GBS serotypes by incorporating machine learning techniques with MALDI-TOF MS to carry out the identification.

**Results:**

In this study, a total of 787 GBS isolates, obtained from three research and teaching hospitals, were analyzed by MALDI-TOF MS, and the serotype of the GBS was determined by a geno-serotyping experiment. The peaks of mass-to-charge ratios were regarded as the attributes to characterize the various serotypes of GBS. Machine learning algorithms, such as support vector machine (SVM) and random forest (RF), were then used to construct predictive models for the five different serotypes (Types Ia, Ib, III, V, and VI). After optimization of feature selection and model generation based on training datasets, the accuracies of the selected models attained 54.9–87.1% for various serotypes based on independent testing data. Specifically, for the major serotypes, namely type III and type VI, the accuracies were 73.9 and 70.4%, respectively.

**Conclusion:**

The proposed models have been adopted to implement a web-based tool (GBSTyper), which is now freely accessible at http://csb.cse.yzu.edu.tw/GBSTyper/, for providing efficient and effective detection of GBS serotypes based on a MALDI-TOF MS spectrum. Overall, this work has demonstrated that the combination of MALDI-TOF MS and machine intelligence could provide a practical means of clinical pathogen testing.

## Background

Group B Streptococcus (GBS), also known as *Streptococcus agalactiae* (*S. agalactiae*), is a gram-positive coccus with a tendency to form chains, and is a beta-hemolytic, catalase-negative, and facultative anaerobe. GBS is the causal pathogen of a wide range of human diseases, including neonatal sepsis, pneumonia, and meningitis [1–4]. In an outbreak of GBS infection or an investigation of GBS infection etiology, strain typing methods including serotyping, geno-serotyping, and multilocus sequence typing method (MLST) are essential for identifying the source of infection and control of infection. GBS serotyping is conducted by latex agglutination (LA) methods based on antibodies specific for capsular polysaccharides (CPSs). There are several known serotypes of GBS such as Ia, Ib, II, III, V, and VI, etc. The serotypes are determined by the distinct structure of the CPSs, which are also important virulence factors leading to human diseases [5–8]. However, if the expression of the CPSs is low, the LA assay may not be successful in strain typing [9]. This restriction could be overcome by genotyping the CPS genes using PCR [10]. Moreover, according to the fragments of seven housekeeping genes, MLST is also a powerful tool for studying the genetic lineages of GBS strains [11]. However, the strain typing methods are costly, time consuming, and laborious. Clinical practitioners would spend extra expense of several tens of USD and tens of hours to days to obtain the strain typing results. It would not meet the clinical needs where strain typing results are urgently required in infection control or outbreak investigation. Consequently, rapid strain typing cannot be fulfilled in clinical practice.

To obtain strain typing results in a more rapid and cost-effective manner, matrix-assisted laser desorption ionization time-of-fly mass spectrometry (MALDI-TOF MS) is a potential tool. Mass spectra are composed of information regarding proteins composition and level. The spectra can be generated by analytical measurement of MALDI-TOF MS. MALDI-TOF MS is widely used in bacterial identification based on the specific protein spectrum in clinical microbiology laboratories [12]. The advantages of this technology are rapid, precise, low cost, and designed for high throughput of samples [13–15]. As a result of these advantages, typing bacteria via specific protein fingerprinting has raised considerable attention. Additional file 1: Table S1 lists previously published work about rapid identification of types of GBS. One of these studies analyzed two highly virulent types of GBS, ST1 and ST17, and disclosed that the *m/z* 6250 and *m/z* 7625 peaks are specific for the two highly virulent types [16]. Another recent study identified *m/z* 6250 and *m/z* 6891 as the specific peaks for serotype VI and III, respectively [17]. Both studies used the ClinPro Tools™ software (Bruker) to perform statistical analyses of mass spectra data from GBS isolates. Normalize all mass spectra to their own total ion count (TIC) and present in a 2-D cluster plot. It was observed that the specific peaks for serotypes. However, a comprehensive pattern for discriminating different types may not be obtained by solely using statistical analysis, partially could be attributed to unperfect reproducibility of MALDI-TOF MS spectra, especially on peak level. A technical review on using MALDI-TOF MS in microbiology revealed that peak-level reproducibility of MALDI-TOF MS spectra is around 90% [18]. Several factors, including type of culture medium, cultivation time, protein extraction process, and inhomogeneities in matrix/analyte-crystals could affect the reproducibility of spectrum [18]. Shifting or drifting of peaks on MALDI-TOF MS spectrum is also a crucial source affecting reproducibility [19, 20]. Peaks appeared in vicinity on MALDI-TOF MS spectra may actually the same peptide ion [21]. However, the peak shifting issue has not yet been well-addressed in previous works. Our team reported that MALDI-TOF MS could be used as the analytical tool for sub-species typing of *Staphylococcus aureus* when using the binning method to cope with the peak shifting issue [21]. In this work, we aimed to evaluate if the binning method is adequate in processing MALDI-TOF MS spectra for geno-serotyping of GBS.

To provide more comprehensive and specific protein patterns for identifying specific strain types, machine learning (ML) is a promising method for analyzing massive and complicated data, such as MALDI-TOF MS spectra [21, 22]. ML is a method of conducting learning from data. Various ML algorithms, including decision tree (DT), and support vector machine (SVM) are robust and widely used algorithms [21]. ML model could learn a specific pattern of data in the training set, and utilize the specific pattern for classification in the testing set [21]. Several studies have demonstrated success in using ML for medical decision fields [21–25].

In this work, we collected hundreds of mass spectra data of various serotypes of GBS. ML models were trained and validated by a robust method to evaluate the classification performance between different ML models. Moreover, informative peak features for discrimination among different serotypes were also selected out. Through the ML models, GBS serotypes could be rapidly detected and may guide adequate management of infectious outbreaks based on existing MALDI-TOF MS data only which exempt extra cost, time, and labor spent on strain typing.

## Result and discussion

### Feature extraction by binning method

Based on the results of previous studies [21, 26], the extent of peak drifting or shifting is ±5 Da. Consequently, bin size ranged from 1-10 Da was tried and evaluated in this study. The template of features (or reference features) could be defined by this method. In Table 1, the number of features under various bin sizes are illustrated. The number of features are larger than 1000 when the bin size is set as 1 Da or 2 Da. In contrast, the number of features can be significantly reduced to several hundred when the bin size is 9 Da or 10 Da. The massive information of MALDI-TOF MS spectra can be condensed into a well-defined combination of features using this approach.

**Table 1:** Number of extracted features among 10 different bin sizes in each model

Bin size (Da)	Number of peaks in each model				
	Type Ia	Type Ib	Type III	Type V	Type VI
1	1621	1621	1622	1621	1620
2	1013	1013	1012	1013	1012
3	779	780	779	779	779
4	644	644	642	643	644
5	576	576	575	576	576
6	493	493	493	493	493
7	468	469	468	469	469
8	428	430	429	429	427
9	398	398	398	397	397
10	380	381	380	380	380

The binning method was used to extract features from MALDI-TOF MS spectra. In this study, we analyzed the region from 2000 m/z to 20,000 m/z, which is the recommended range to be used in the clinical microbiology laboratory [27]. The binning method was used here to address the drift or shift phenomenon of peaks in MS spectra. Moreover, the dimension of features can be considerably reduced by using the binning method. Dimension reduction is crucial in applying ML algorithms, especially when a relatively small dataset is used. The optimal bin size was systemically evaluated in this study. When the bin size is large, too many peaks would be coalesced into a single bin and the processed data may not be sufficient for classifying different serotypes. In contrast, when bin size is narrow, performance of classification may be compromised because of Hughes phenomenon [28].

### Overview of the training data sets

To visualize the whole mass spectrum of GBS, we presented them in so-called pseudo gel views, which were generated from the pre-processed mass spectra. In the pseudo gel views (Additional file 1: Figure S1) the intensities are gray-scaled. The abscissa indicates the mass-to-charge ratio (m/z), and the mass range of m/z is 2000–10,000 because of the resolution of the picture. The ordinate indicates the mass spectra. Additional file 1: Figure S1 is the pseudo gel view of 324 mass spectra of GBS. The pseudo gel can be divided into five blocks based on the serotypes, from top to bottom followed by serotype Ia, Ib, II, III, V, and VI. Some of the discriminative peaks could be observed by the naked eye through this approach. For example, signals over *m/z* 6250 and *m/z* 6890 are frequently observed in serotype VI, and signals over *m/z* 2960 and *m/z* 6890 are discriminative peaks for serotype III. However, the disadvantages of the pseudo gel are explicit, though it is a commonly used approach. First, the method fails to identify discriminative peaks when the intensity of peaks is not high enough. Second, observation by the naked eye depends on well-trained staff and is a highly operator-dependent method, where reproducibility may be compromised. Third, the difference of MS spectra is subtle. Finding subtle differences from a complicated MS spectrum is time-consuming and labor-intensive. To conduct a more reliable analysis of MS spectra and obtain a more robust result, introducing data mining techniques, including ML methods, is a promising and inevitable choice.

**Fig. 1:**
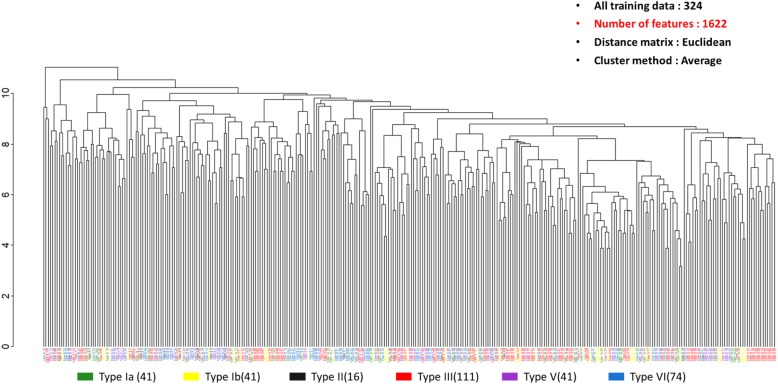
Flowchart of GBS serotype prediction in this work. The study can be divided into three parts: data collection, data analysis, and prediction analysis

**Fig. 2:**
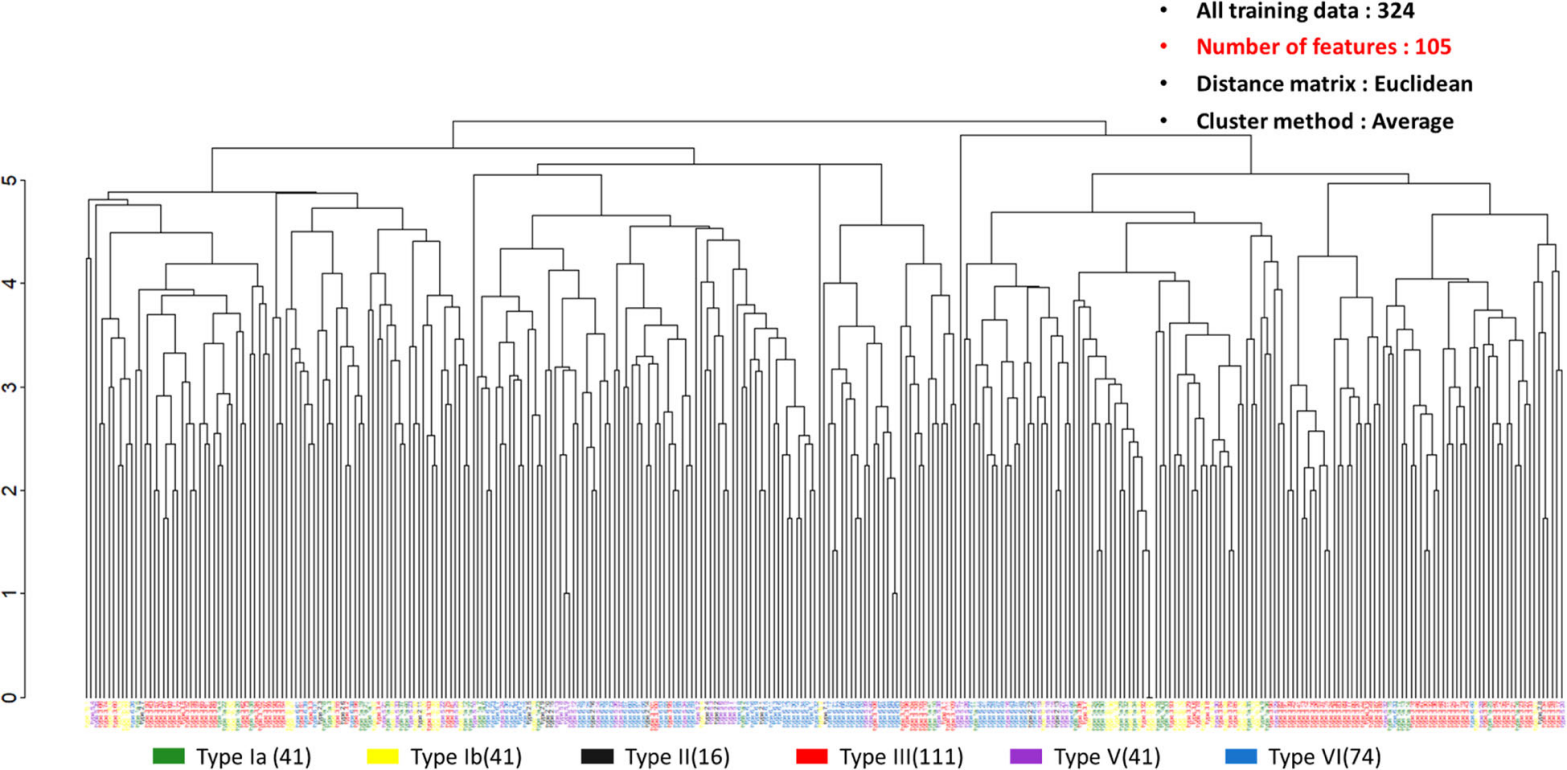
Example of binning method for feature extraction. At the upper part, we stacked three mass spectra as examples, and their ranges are from *m/z* 2000 to *m/z* 2030. At the lower part, all mass spectra were divided by using ten different sizes of error regions from 1 Da to 10 Da. The blue squares indicate that at least one peak from any data is in the range of the bin

UHCA is a purely data-driven classification method. This method could be used to illustrate the efficacy of the binning method. In Fig. 1, the bin size is set to 1 Da, where a total of 1622 features are used. GBS isolates with the same serotypes are distributed diversely. Specifically, the main serotypes (i.e. serotype III and VI) were found to be divided into several subgroups. In contrast, in Fig. 2, we collected 105 important features from the predictive models of five serotypes. The clusters of GBS serotypes are relatively explicit. Through UHCA, the different serotypes of GBS in the training dataset were illustrated to be preliminarily grouped together when an adequate bin size was selected for data preprocessing.

### Feature selection by one rule (OneR) and Pearson correlation coefficient (PCC)

Features were extracted by the binning method and ranked by OneR and PCC. The forward selection method was then used to explore the best combination of features. Additional file 1: Figure S2 (a)~(j) show the trends of accuracies of the predictive models with different bin sizes, feature number, and ML algorithms when using OneR for feature selection. By the systemic assessments, the optimal bin size value and the best feature combinations were determined. Briefly, the results showed that accuracies of predictive models were optimized by setting bin sizes as 9 Da, 7 Da, 9 Da, 1 Da, and 9 Da for serotype Ia, Ib, III, V, and VI, respectively. PCC was used as the other feature selection method. The trends of accuracies of the predictive models with different bin sizes, features number, and ML algorithms by using PCC were illustrated in Additional file 1: Figure S3 (a)~(j). The results showed that the accuracies of predictive models were optimized by setting bin sizes as 9 Da, 3 Da, 6 Da, 9 Da, and 7 Da for serotype Ia, Ib, III, V, and VI, respectively. Based on these bin sizes, the most discriminative peaks (top 10) selected by OneR and PCC were illustrated in Additional file 1: Tables S3 and S4, respectively.

We also performed more than 5-fold cross validations (10 to 30) to estimate the robustness of the prediction. To evaluate the 5-fold and (10–30)-fold cross validations, we ran the cross validations for each selected bin size/number of features prediction. And, in order to conveniently compare the results of cross validations with different folds, we presented them as ROC curves. The results revealed that the performance of five-fold and more than five-fold cross validation is similar. Additional file 1: Figure S4 (a)~(j) and S5 (a)~(j) show the ROC curves.

### Evaluating the predictive models by cross-validation and independent dataset

Predictive models of the five serotypes were trained and validated using the optimal bin size and features determined in the step of feature selection. The performance of the predictive models was evaluated by 5-fold cross validation. The models with highest performance were selected as the final models which were further validated by independent testing dataset.

The predictive models with the highest performance for each serotype are shown in the Tables 2 and 3, containing the details of bin size, feature number, Sn, Sp, Acc, and MCC. Briefly, the predictive models attained higher performance using PCC as the feature selection method than OneR. For most predictive models, the optimal bin size was 9 Da (Table 3), which is consistent with the findings of previous studies reporting the extent of peak drifting or shifting as ±5 Da [21, 26]. Both algorithms (i.e. random forest and SVM) showed similar performance. The predictions of the models were fairly balanced regarding the Sn, Sp, and MCC.

**Table 2:** Performance (five-fold cross validation) of the predictive models for each serotype when using OneR for feature selection. SVM: Support Vector Machine; Sn: Sensitivity; Sp: Specificity; Acc: Accuracy; MCC: Matthews Correlation Coefficient

Serotype	Feature selection	Classifiers	Bin size	Number of features	Sn	Sp	Acc	MCC
Ia	OneR	Random Forest	9	42	95.1%	89.1%	90.9%	0.804
		SVM	9	38	93.5%	91.9%	92.4%	0.828
Ib		Random Forest	7	48	83.7%	81.0%	81.8%	0.611
		SVM	7	46	76.4%	74.3%	74.9%	0.473
III		Random Forest	9	28	90.1%	87.4%	88.3%	0.753
		SVM	9	16	86.5%	85.5%	85.8%	0.700
V		Random Forest	1	43	86.2%	85.9%	86.0%	0.690
		SVM	1	43	81.3%	81.0%	81.1%	0.590
VI		Random Forest	3	47	93.2%	91.6%	92.2%	0.837
		SVM	9	13	91.2%	89.6%	90.2%	0.796

**Table 3:** Performance (five-fold cross validation) of the predictive models for each serotype when using PCC for feature selection. SVM: Support Vector Machine; Sn: Sensitivity; Sp: Specificity; Acc: Accuracy; MCC: Matthews Correlation Coefficient

Serotype	Feature selection	Classifiers	Bin size	Number of features	Sn	Sp	Acc	MCC
I a	PCC	Random Forest	9	35	100.0%	96.1%	97.3%	0.939
		SVM	9	28	100.0%	99.6%	99.8%	0.994
I b		Random Forest	3	38	100.0%	93.3%	95.3%	0.899
		SVM	3	30	100.0%	99.3%	99.5%	0.988
**III**		Random Forest	6	7	91.0%	90.2%	90.5%	0.795
		SVM	6	7	91.0%	89.7%	90.2%	0.789
**V**		Random Forest	9	46	100.0%	92.3%	94.6%	0.885
		SVM	9	43	100.0%	99.6%	99.8%	0.994
**VI**		Random Forest	6	31	97.3%	91.6%	93.7%	0.872
		SVM	7	18	94.6%	93.2%	93.7%	0.868

In the external validation using the independent dataset, the performance of all the models declined. The decrease in prediction performance could be caused by the small sample size used in the study, especially for serotype Ia, Ib, and V (Table 4). The prediction performance would be compromised when the training data cannot offer rich information of classification. In contrast, for the major serotypes, namely serotypes III and VI, the performance of predictive models declined less and remained balanced in the external validation (Table 5). More samples should be used for training more robust ML models in order to apply them into clinical practice in the future.

**Table 4:** Characteristics of the training data sets of each serotype after oversampling

Models of serotypes	Type of data	Original data	Post oversampling	Total
Ia	Ia	41	123	407
	Non-Ia	284	284	
Ib	Ib	41	123	407
	Non-Ib	284	284	
III	III	111	111	325
	Non-III	214	214	
V	V	41	123	407
	Non-V	284	284	
VI	VI	74	148	399
	Non-VI	251	251

**Table 5:** Performance of the predictive models for each serotype by using independent testing data. PCC was used as the feature selection method. SVM: Support Vector Machine; Sn: Sensitivity; Sp: Specificity; Acc: Accuracy; MCC: Matthews Correlation Coefficient

Serotype	Bin size of Peaks	Number of features	Classifiers	Sn	Sp	Acc	MCC
**I** a	9	28	Random Forest	66.0%	61.4%	61.9%	0.168
			SVM	19.1%	94.9%	87.1%	0.172
I b	3	30	Random Forest	55.6%	54.8%	54.9%	0.071
			SVM	54.0%	38.9%	41.0%	−0.050
**III**	6	7	Random Forest	73.0%	74.1%	73.9%	0.405
			SVM	68.0%	71.3%	70.6%	0.336
**V**	9	43	Random Forest	63.6%	40.6%	43.4%	0.028
			SVM	5.5%	94.1%	83.4%	−0.007
**VI**	7	18	Random Forest	70.4%	70.3%	70.4%	0.381
			SVM	67.6%	64.4%	65.4%	0.297

### Informative peaks in each predictive model

Additional file 1: Tables S5, S6, S7, S8, and S9 show the distributions of discriminative features in each model. The listed features were selected by PCC. Some of the informative peaks found in this study, such as *m/z* 6251 [17], *m/z* 6891 [17], and *m/z* 7620 [16], were reported as characteristic peaks for serotypes VI, Ib/III, and III, respectively. Specifically, *m/z* 6251 was purified and identified as CsbD like protein (gi: 445998854 and gi:77413040) [17, 29]. The peak at *m/z* 6891 was identified as UPF0337 protein gbs0600 belonging to the UPF0337 (CsbD) family [17]. The peak at *m/z* 7620 was reported as the small subunit of exodeoxyribonuclease VII (Gi: 77409335) [29]. In this study, we focused on using ML approach to classify various serotypes of GBS by recognizing specific peaks composition (i.e., the pattern of spectra). In the current stage, we have validated that the ML approach is workable by robust validation methods, including cross-validation and external validation. On the basis of the peak composition, ML could be promising for determining essential peptides of drug resistance by revealing the identities of the peaks in the future.

The peak at *m/z* 6891 was reported as a characteristic feature for GBS serotypes Ib and III [17]. In contrast, in this study, *m/z* 6891 was selected as the informative feature for all of the predictive models. The frequency of occurrence of *m/z* 6891 was higher in serotypes Ia, Ib, and III, but lower in serotypes V and VI (Additional file 1: Tables S5, S6, S7, S8, and S9). Though the frequency of occurrence of the selected peaks showed considerable difference among the binary classes, the peaks did not present only in one class. For example, the peaks at *m/z* 7616~7624.9, *m/z* 6248~6256.9, and *m/z* 6887~6895.9 were all selected as the informative peaks for both predictive models of serotype Ia and serotype V, but the distribution of these three peaks was different among serotype Ia and serotype V: the higher frequency of occurrence of *m/z* 6887~6895.9 and lower frequency of occurrence of *m/z* 7616~7624.9 and *m/z* 6248~6256.9 was characteristic for serotype Ia. In contrast, the higher frequency of occurrence of *m/z* 6248~6256.9 and lower frequency of occurrence of *m/z* 7616~7624.9 and *m/z* 6887~6895.9 was characteristic for serotype V. Briefly, the whole pattern of multiple informative peaks but not a single peak should be taken as the features in predicting or classifying the serotypes.

### Peak pairs of the binary predictive models

**Fig. 3:**
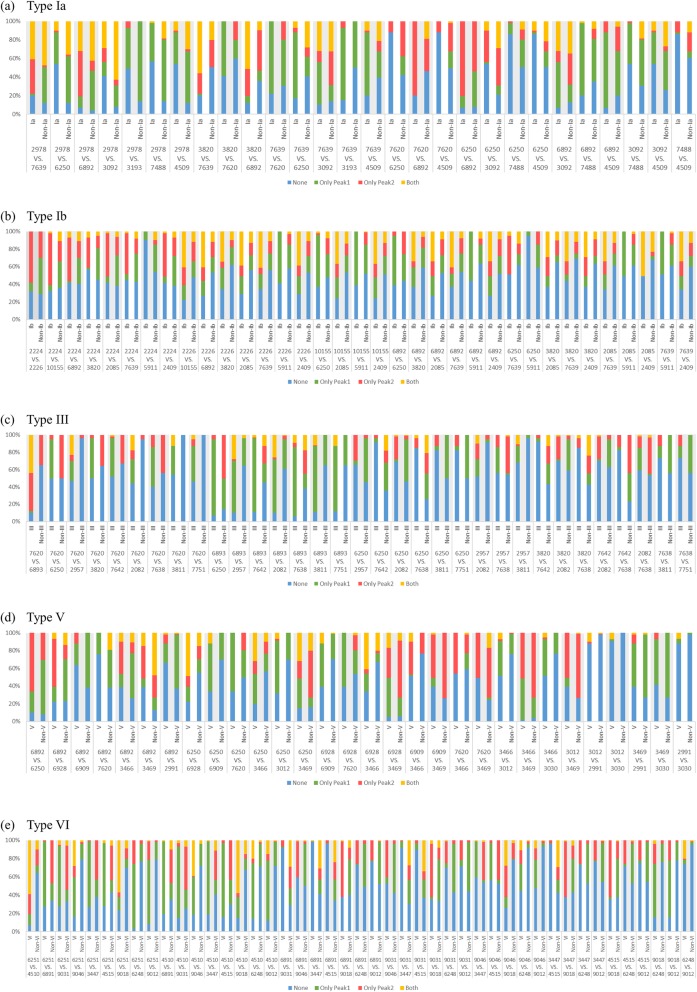
Example of a way to find the main peak. Following Fig. 2, we took the bin size 5 Da (from *m/z* 2010 to *m/z* 2014.999) for an example

The fingerprint of multiple features is thought to provide more comprehensive information than a single feature [23]. However, the whole pattern of features is difficult to be fully explained together. To address this issue, the concept of a pair of essential features was proposed and used in this study. A peak pair was generated by iteratively pairing the top 10 essential peaks which had been selected by PCC. The importance of these peak pairs was examined by chi-square. The peak pair can provide part of the features pattern, and the results are relatively easily to interpret. The patterns of co-presence, co-absence, and presence-absence of essential peaks can be illustrated by this approach. Regarding the most predominant serotype (i.e., serotype III) (Table 6), *m/z* 6251 [17], *m/z* 6891 [17], and *m/z* 7620 [16] were reported as characteristic peaks and also found as informative features in this study. Three pairs could be generated by these three peaks, namely pair 1 (*m/z* 7620 & *m/z* 6893), pair 2 (*m/z* 7620 & *m/z* 6250), and pair 10 (*m/z* 6893 & *m/z* 6250) (Fig. 3c). Some interesting patterns of the peak pairs can facilitate classifying serotyping rapidly. For example, regarding pair 2, when only *m/z* 6250 is present and *m/z* 7620 is absent, a GBS isolate can be predicted as non-serotype III with high probability. Additionally, regarding pair 10, a GBS isolate can be predicted as serotype III with high confidence when *m/z* 6893 is present and *m/z* 6250 is absent. Furthermore, besides facilitating rapid prediction and classification, the patterns of peak pairs may also provide some valuable hints for further investigation in the future. For example, the distinct pattern of the peak pair of serotype III revealed that the presence of *m/z* 6893 and absence of *m/z* 6250 may imply a specific relationship between *m/z* 6893 and *m/z* 6250. Peaks of *m/z* 6893 and *m/z* 6250 represent UPF0337 protein gbs0600 [17] and CsbD like protein [29], respectively. Both these proteins are similar to the CsbD protein, which is thought to be a stress response protein and may contribute to the virulence of GBS [30]. It was reported that the difference between *m/z* 6893 and *m/z* 6250 is seven amino acids [17]. The peak at *m/z* 6893 found for most GBS serotype III may be the result of the *m/z* 6250 serotype modified though the infection process of prophage Lambda SA03 [17]. In conclusion, the specific patterns of peak pairs not only provide keys for rapid classification, they also reveal many hints which may be related to the virulence or evolution of pathogens.

**Table 6:** Data statistics of the training data set and independent testing data set among each serotype of GBS (serotype Ia, Ib, II, III, IV, V, VI, VII, and unknown serotypes)

Serotypes	Number of Mass Spectra (%)^a^	
	Training Data Set	Independent Testing Data Set
Ia	41 (12.6%)	47 (10.2%)
Ib	41 (12.6%)	63 (13.6%)
II	16 (4.9%)	44 (9.5%)
III	111 (34.2%)	100 (21.6%)
IV	–	5 (1.1%)
V	41 (12.6%)	55 (11.9%)
VI	74 (22.8%)	143 (31.0%)
VII	1 (0.3%)	2 (0.4%)
VIII	–	1 (0.2%)
unknown	–	2 (0.4%)
Total	325 (100%)	462 (100%)

a: Percentage of each serotype in two kinds of data sets

### Web tool implementation

**Fig. 4:**
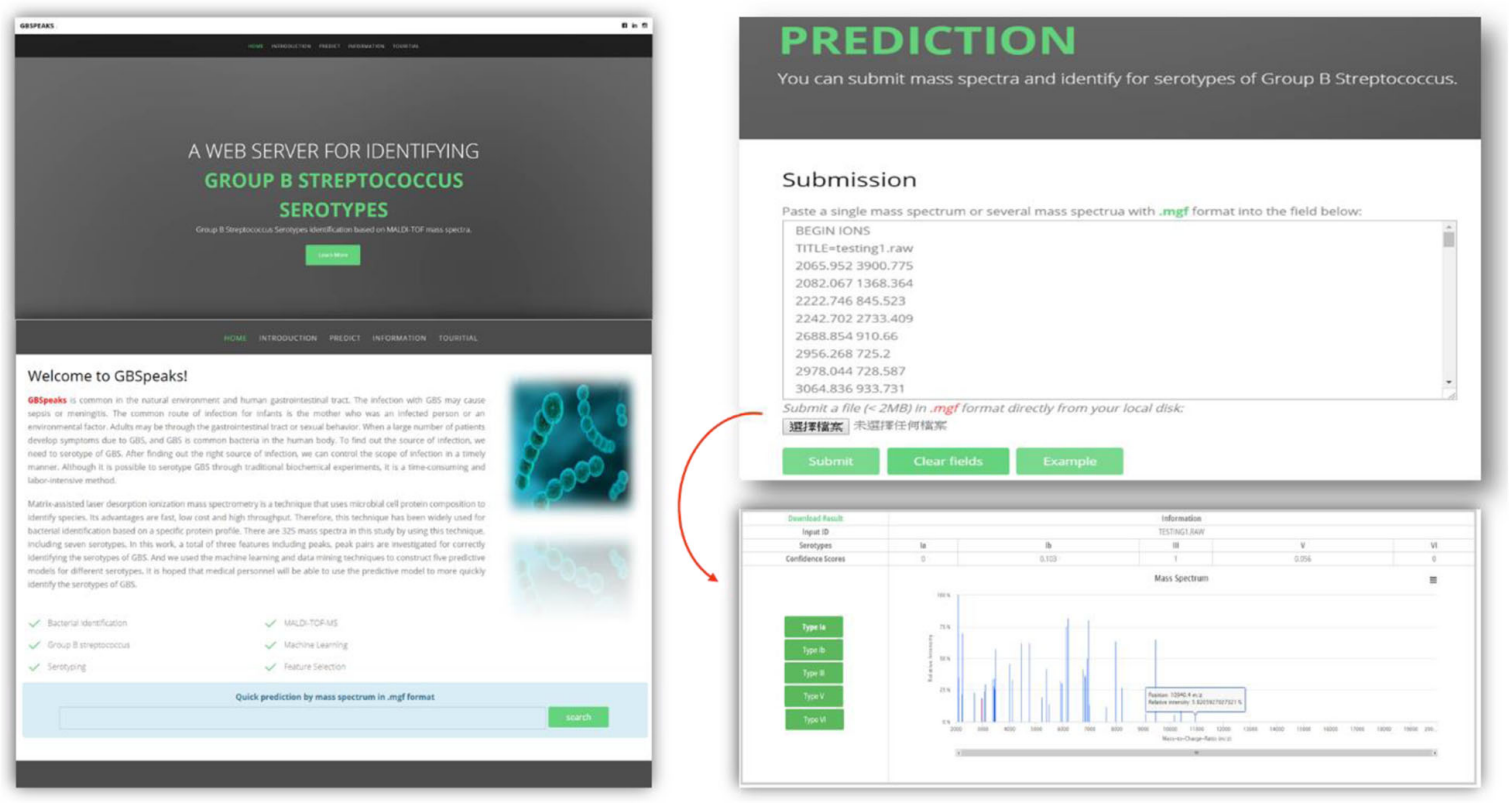
Example of peak pair. When the *p*-value was less than or equal to 0.05, this peak pair was statistically significant

To enhance application of the ML models to clinical practice, we constructed a web tool named GBSTyper. The specific functions of the website are to provide: 1) descriptive statistics and visualization of the input data; 2) informative combinations of features for different GBS serotypes; and 3) prediction of GBS serotype using multiple ML predictive models. Academic researchers and clinical practitioners can use the web tool to enhance either their studies or rapid strain typing in the investigation of an outbreak. Figure 4 illustrates the web tool.

## Conclusion

This study described a tool for rapid classification of GBS serotypes based on MALDI-TOF MS spectra. The binning method and PCC were used for processing the MALDI-TOF MS spectra data. Informative peaks, and peak pairs were found through this approach. These essential peaks and specific relationship of peaks (i.e. peak pairs) may provide hints for further investigation. For clinical practice, the predictive ML models of serotypes were constructed and validated by an independent testing dataset. GBS serotypes could be rapidly predicted by the models at a low cost. Moreover, a web-based tool was built to enhance the application of the results (http://csb.cse.yzu.edu.tw/GBSTyper/).

## Methods

**Fig. 5:**
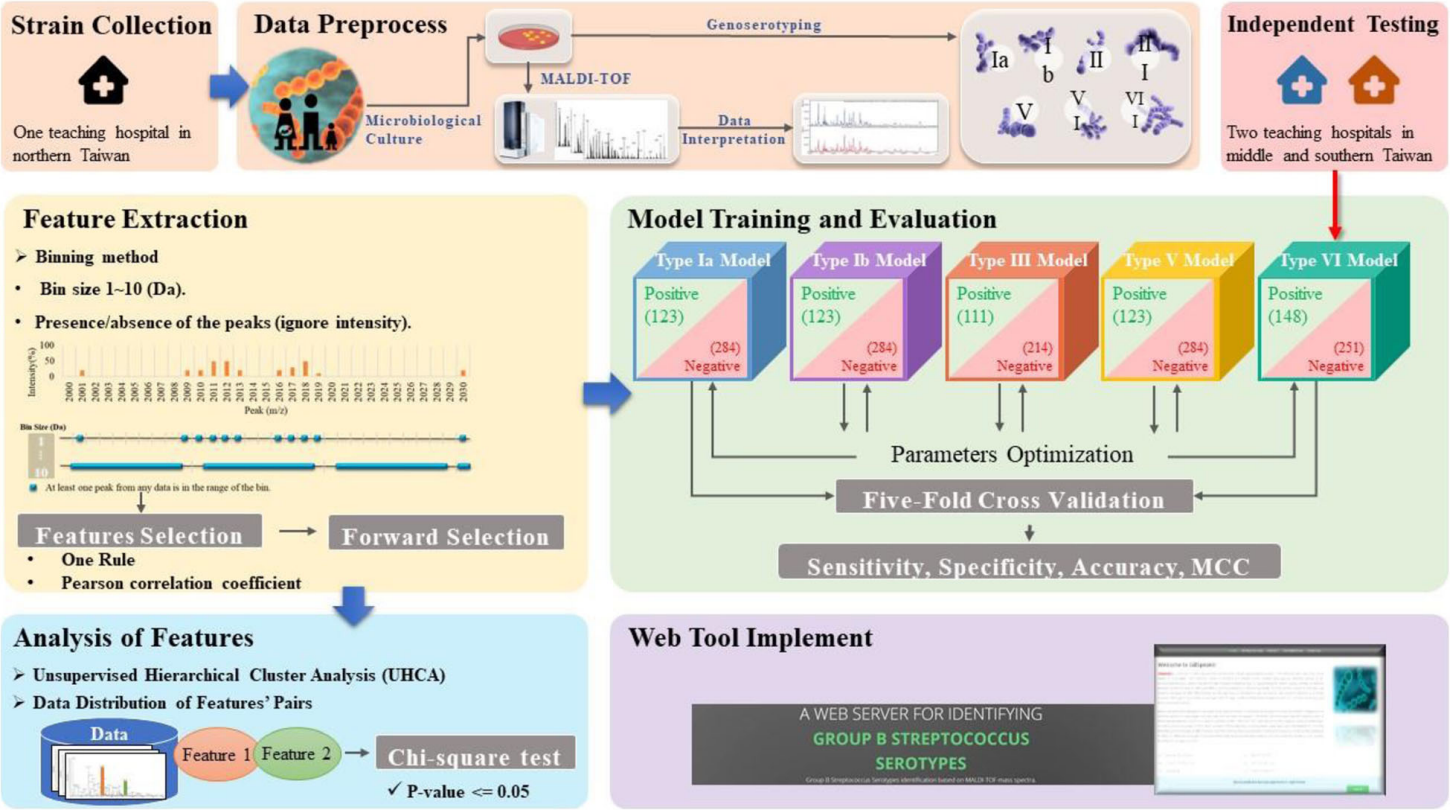
Example of the feature selection method, OneR. There are six data as examples in the left Table. (3 Type III and 3 Non-type III). In the right table, the statistics of the number of occurrences in Peak 1 and Peak 2 are counted. The red words in the table are the numbers of judgment errors

The study can be divided into three parts: data collection, data analysis, and prediction analysis. Data collection: training data set samples were obtained from the bacterial bank in one teaching hospital in northern Taiwan. Independent testing data set samples were obtained from the bacterial bank of the other two teaching hospitals in middle and southern Taiwan. MALDI-TOF MS was used to obtain the mass spectra, and the samples were typed by geno-serotyping. Data analysis: we used the binning method to extract features from the original mass spectra. Feature selection was conducted by two different feature selection algorithms to identify the important features of each serotype. Prediction analysis: We designed predictive models using the selected features of each serotype by two ML algorithms. The models were tested and evaluated by five-fold cross-validation. Finally, we used the independent testing data set for external validation of the models. The overall study flow is presented in Fig. 5.

### MALDI-TOF MS experiments

All the GBS isolates used in this study were collected as part of routine procedures in the clinical microbiology laboratories of the tertiary centers. Patient identities were removed before storage in bacterial banks. All the isolates were stored in bacterial banks until being used for further analyses. Only characteristics of GBS were recorded, informed consent or ethical approval was not necessary for the study [31]. The isolates were stored at − 70 °C until use. The fresh GBS colonies that grew on BBL™ Trypticase™ Soy Agar with 5% Sheep Blood (TSA II) (Becton Dickinson, MD, USA) for 24 h were picked and smeared onto a MALDI target plate in thin films. A 1 μl aliquot of the α-cyano-4-hydroxycinnamic acid (CHCA) matrix solution [saturated, 50% acetonitrile (CAN)/ 2.5% trifluoroacetic acid (TFA)] was overlaid on the sample spot of the MALDI-target plate. The sample-matrix was dried at room temperature before analyzing by mass spectrometry to obtain the data. The mass spectra were acquired in the mass range of 2000 to 20,000 m/z in linear mode by MALDI-TOF (Bruker Daltonik GmbH, Leipzig, Germany). The 240 laser shots of each sample were collected and companied with test standard (BTS) (part no. 255343, Bruker Daltonik GmbH, Leipzig, Germany) as calibration and control with a linear positive model to analyze each time. The results of the mass spectrum were aligned to the database and the scores were calculated using MALDI Biotyper 3.1 software (Bruker Daltonik GmbH, Bremen, Germany). Scores of more than 2 underwent peak signal analysis.

Prior to a further analysis of peak values, the MS raw data should be transformed into a Microsoft excel file by the embedded function of FlexAnalysis 3.3 (Bruker Daltonik GmbH, Bremen, Germany). In this work, the original MS signals were smoothed by Savitzky-Golay algorithm and their baselines were subtracted by the top hat method. Meanwhile, some thresholds that were adopted to extract reasonable peaks were set as follows: signal-to-noise ratio was two, relative intensity and minimum intensity were both zeros, the maximal number of peaks was 200, peak width was six, and the height was 80%. After performing FlexAnalysis 3.3 along with the specified parameters, the Microsoft excel file, including two columns of ions m/z and intensity values, was obtained.

### Preparation of training and independent testing datasets

A total of 325 GBS training mass spectra data sets were collected by Linkou Chang Gung Memorial Hospital, of which 225 were isolated from blood of newborns (*n* = 127) and non-pregnant adults (*n* = 98) from 2003 to 2014. The other 100 GBS isolates were obtained from vaginal specimens of pregnant women (35–37 weeks) from 2016. The total number of complete data sets included in this study is given in Table 6.

In addition, a total of 462 GBS mass spectra were used as the independent testing data set. Among them, 104 data sets were obtained from 98 patients from June 2007 to October 2010. Ninety-five invasive strains were isolated from blood of children (*n* = 11) and non-pregnant adults (*n* = 78) and 9 colonizing strains from the vagina of pregnant women at China Medical University Hospital in central Taiwan. The remaining 358 data sets were obtained by Linkou Chang Gung Memorial Hospital. The number of complete data set included in this study is given in Table 6.

Single colonies recovered from BBL™ Trypticase™ Soy Agar with 5% Sheep Blood (TSA II) were used for geno-serotyping. Briefly, total bacterial DNA was purified and a set of multiplex PCR was conducted. The PCR products were analyzed by electrophoresis and the characteristic bands pattern on the electrophoresis gel would designate specific serotype for the bacterial isolate. We followed the methods described in previous works [32, 33].

For the data sets of certain serotypes (e.g. serotype II and VII) that were relatively small, predictive models were not constructed. To construct models for classifying the five different serotypes of GBS, the one-against-all strategy, constructing five binary models for the five serotypes, was adopted in this study. For constructing predictive models for serotypes Ia, Ib, V, and VI, the oversampling method was used to increase the number of the minor class to address the imbalance issue for these serotypes. Oversampling minority class samples three times was adopted for preparing training datasets of serotype Ia, Ib, and V; for training datasets of serotype VI, the minority class samples were oversampled twice (Table 4).

### Feature extraction

**Fig. 6:**
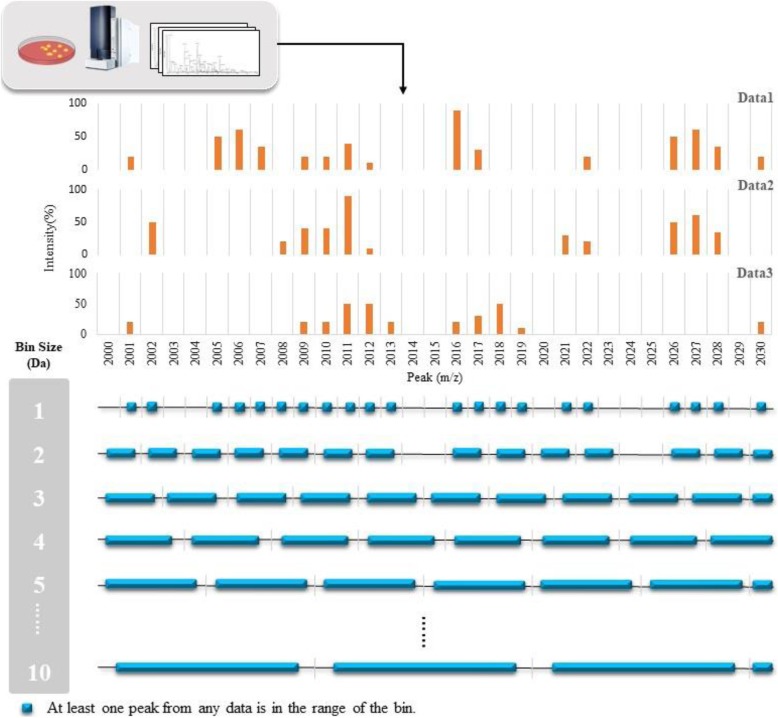
Data distribution of training data set by unsupervised hierarchical cluster analysis (UHCA) with bin size 1 Da

**Fig. 7:**
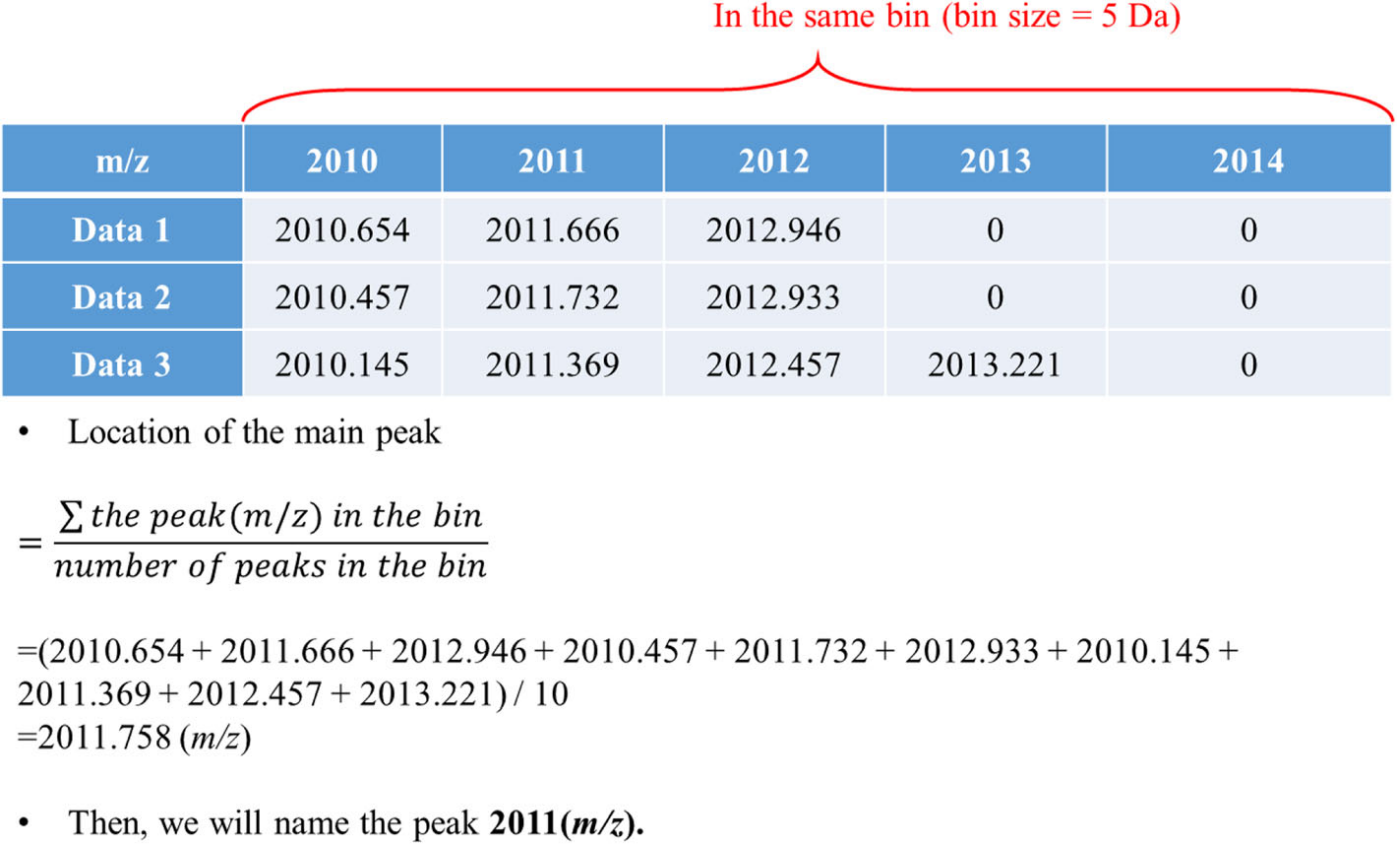
Data distribution of training data set by UHCA with features selected from the five models

The binning method (or bucketing method) was used for constructing the templates of the serotypes in this study. A peak shift or drift in MALDI-TOF MS spectra has been observed in several studies [21, 34], which means a specific peptide may not always appear at the same mass-to-charge (m/z) values of a MS spectrum. A method of building a serotype-specific template is useful for extracting and defining certain combinations of features from MALDI-TOF MS spectra [21]. A well-defined combination of features (i.e. a template) is crucial for operating techniques of ML. The binning method is one of the most commonly used preprocess techniques in MS data analysis. The main goal of the method is to preserve the information of the raw data, while reducing the dimensions to facilitate subsequent processing and mining phases [35]. Figure 6 shows three mass spectra as an example at the upper part, ranging from *m/z* 2000 to *m/z* 2030. At the lower part, all mass spectra were divided by ten different sizes of bins (from 1 Da to 10 Da) to evaluate the optimal value of bin size. If there are blue squares in the bins, it means that there is at least one peak in the range of the bin. Adjacent peaks would be coalesced into a single bin. Features extraction (peaks finding) could be accomplished using this method. Moreover, the representative m/z value of a bin was determined for defining a single feature of the template. An example is illustrated in Fig. 7. We took a bin from Fig. 6, and its size is 5 Da ranging from m/z 2010 to m/z 2014.999. All peaks in the range of each data are recorded in the table. If it is 0, it means there is no peak. In this study, only the information of peak presence, or absence, was employed while the peak intensity information was omitted [21].

### Pairwise peaks analysis

**Fig. 8:**
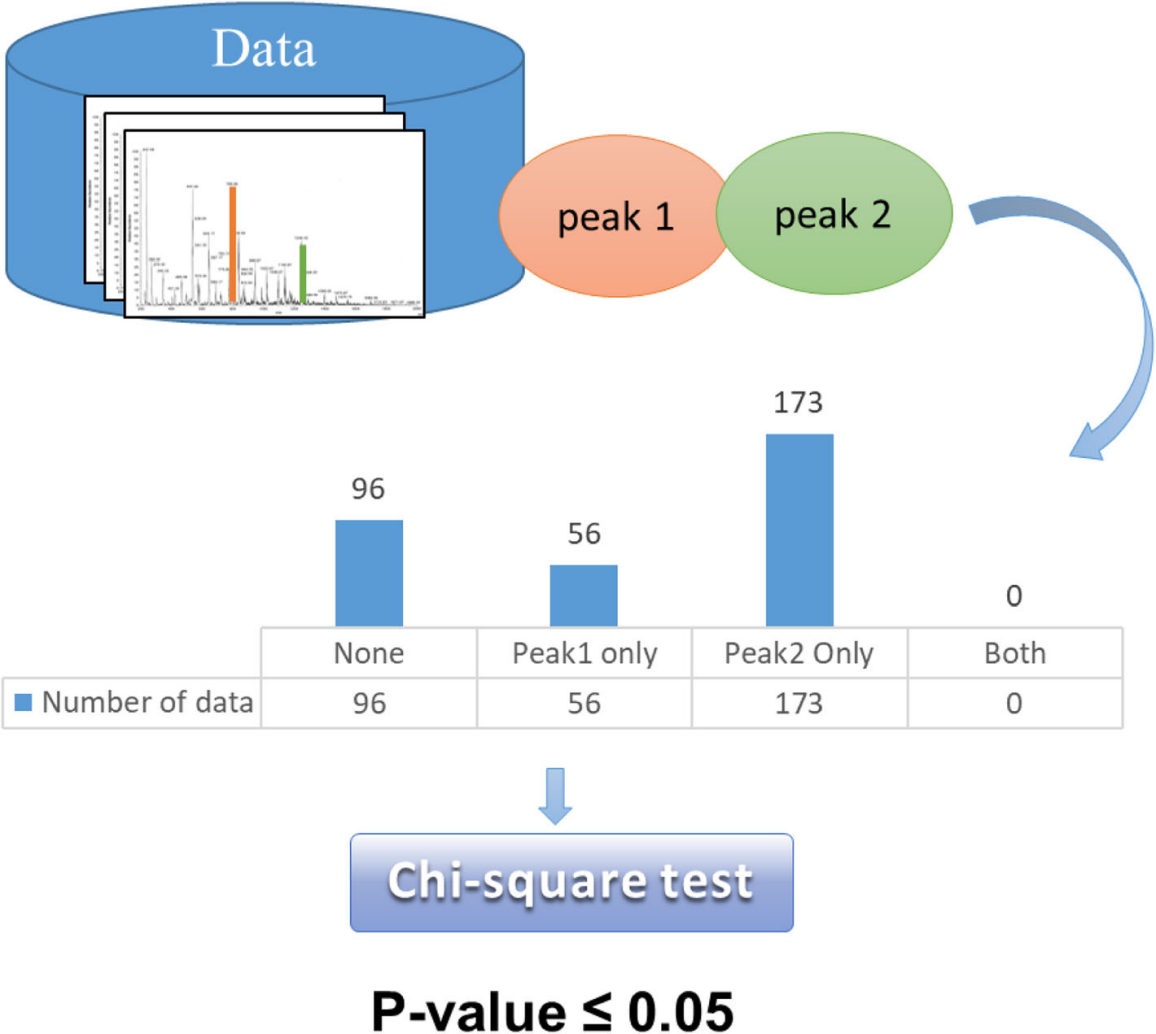
Data distribution of discriminative peak pairs for each serotype. The peaks were selected and ranked by PCC. **a** Type Ia, (**b**) Type Ib, (**c**) Type III, (**d**) Type V, and (**e**) Type VI. The distribution of data for each pair of the training data set in each model is shown. The term ‘none’ in the legend indicates that both peaks are absent. The terms ‘peak1’ and ‘peak2’ represented that only the peak above VS appeared or the lower peak appeared. The term ‘both’ represented the simultaneous appearance of two peaks. Overall, there was a significant difference of each pair based on different combinations in the positive and negative data set

After features were extracted from 2 kDa to 20 kDa by the binning method, the top ten features in the ranking list of the Pearson correlation coefficient (PCC) were used. The ten peaks were randomly paired. That is, each peak pair contains two peaks, and a total of seventy-two combinations. There were four possible relationships for each pairwise peaks (both peaks present, both peaks absent, and only one peak present). Chi-square was used to evaluate the relationship between the peak pairs and each serotype. When the *p*-value was less than or equal to 0.05, the peak pair was considered a statistically significant characteristic for the serotype. Figure 8 shows an example of a peak pair. Assume that Peak 1 and Peak 2 are two of the top ten features of the data set ranked by PCC. The table in the figure is the amount of data that counts the four relationships of the peak pair.

### Feature selection methods

We used two different algorithms, One Rule (OneR) Feature Evaluation and PCC, to pick out meaningful features for GBS typing. Both algorithms can be used to assess the importance of the features. All the features were scored and sorted according to the OneR or PCC algorithm, and forward selection was used for evaluation of the performance of a specific combination of features. The performance evaluation was conducted in WEKA, by five-fold cross-validation. WEKA was used as the main analyzing tool in this study [36]. It is a software for data exploration written in Java, developed at the University of Waikato, New Zealand. It is a set of software that provided various tools for data mining and machine learning, including data pre-processing tools, classification tools, etc., can also be presented in visual form.

OneR is a rule-based strategy to evaluate the classifying ability for each attribute. In this investigation, each attribute was regarded as a single rule for classifying between, for instance, type III samples and non-type III samples. OneR was learned as a one-level decision tree to generates a set of rules that test one particular attribute [37]. There were mainly three steps contained in the OneR investigation of each attribute:

1. *Two branches for the attribute’s values (1 and 0)*
2. *Each branch will be assigned a class label (ex. Type III or non-type III) with highest frequency*
3. *Calculation of error rate for each branch: proportion of samples that don’t belong to the assigned class of their corresponding branch*

**Fig. 9:**
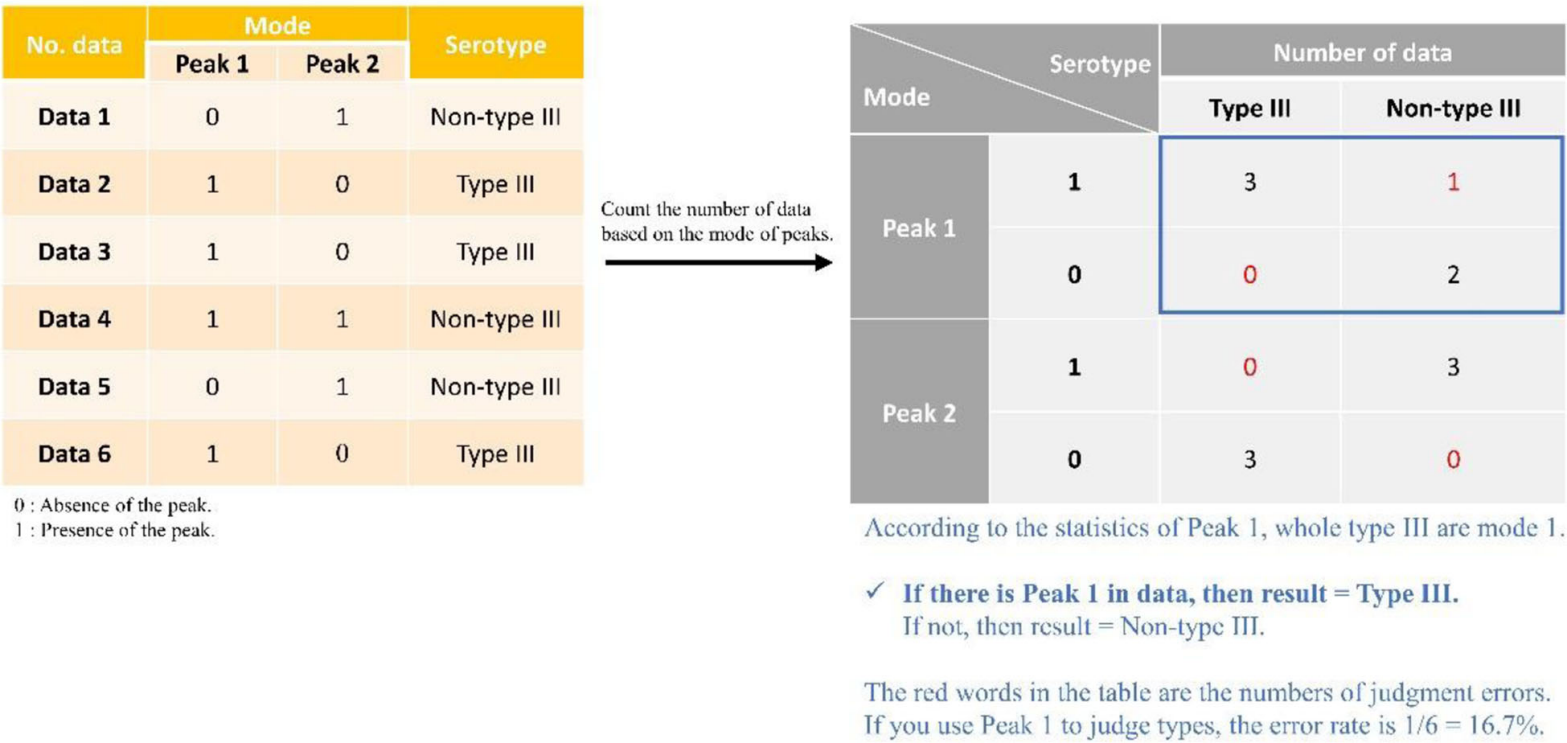
Prediction page of the GBS Website

After the calculation of error rate against all attributes, all of them were ranked according to the error rate in ascending order. The attribute containing lowest error rate represents a best classifying ability. Figure 9 shows an example that there are two different and independent features: peak1, and peak2 would affect the result (type III & non-type III). All features were analyzed by this feature selection method. For each feature, there are two conditions: 1 (presence) or 0 (absence). The rule for peak1 is decided based on the relationship between presence/absence of peak1 and the serotypes. As demonstrated in Fig. 9, presence of peak1 is highly related to non-type III serotypes; in contrast, absence of peak1 is highly correlated to type III serotype. The importance of the features would be ranked according to the performance following the rules.

PCC is a metric used to detect the linear dependence (correlation) between two variables *X* and *Y* by generating a value between − 1 and + 1:

$$ \rho \left(X,Y\right)=\frac{\operatorname{cov}\left(X,Y\right)}{\sigma_X{\sigma}_Y}=\frac{E\left[\left(X-{\mu}_X\right)\left(Y-{\mu}_Y\right)\right]}{\sigma_X{\sigma}_Y} $$

in which cov(*X*,*Y*) is the covariance between *X* and *Y*, and *σ*_*x*_/*σ*_*y*_ is the standard deviation of *X*/*Y*. For instance, the type III samples are labeled as + 1 while those non-type III samples are labeled as − 1. To calculate the PCC value for a given attribute (peak), the samples with/without this attribute shall be labeled as 1/0. In the investigation of correlation between a given attribute and the sample distribution, a higher PCC value indicates that the evaluated attribute has a higher correlation to the distribution of positive and negative samples.

### Unsupervised hierarchical cluster analysis (UHCA)

In this study, UHCA was carried out using the Euclidean distances obtained between mass spectra. For UHCA, several of the most relevant peaks taken from the mass range of *m/z* 2000–20,000 were used. The average linkage was employed as the clustering method [38]. UHCA was performed with R software.

### Support vector machine

This study was involved in the one-against-all multi-class classification among five GBS serotypes. For instance, the type III samples (positive training data) and non-type III samples (negative training data) were labeled with + 1 and − 1, respectively. The training dataset is *X* = {*x*^*t*^, *c*^*t*^} where *c*^*t*^ = + 1 if *x*^*t*^ ∈ positive dataset and *c*^*t*^ = − 1 if *x*^*t*^ ∈ negative dataset. This work wants to identify *w* and *w*_0_ such that

$$ {w}^T{x}^t+{w}_0\ge +1\;\mathrm{for}\;{c}^t=+1\;\mathrm{and}\;{w}^T{x}^t+{w}_0\le -1\;\mathrm{for}\;{c}^t=-1 $$

which can be rewritten as

$$ {c}^t\left({w}^T{x}^t+{w}_0\right)\ge +1. $$

This problem could be induced to find out an optimal separating hyperplane that can maximize the margin between two classes [39]. The distance of *x*^*t*^ to the discriminating hyperplane is

$$ \frac{\mid {w}^T{x}^t+{w}_0\mid }{\left\Vert w\right\Vert} $$

and we would like the distance to be higher than a specific value *h*:

$$ \frac{c^t\left({w}^T{x}^t+{w}_0\right)}{\left\Vert w\right\Vert}\ge h,\forall t\; and\;{c}^t\in \left\{+1,-1\right\}. $$

The support vector machine (SVM) was an advanced algorithm used to identify a hyperplane between two classes with maximum margin based on n-dimensional vector space [39]. With an attempt to maximize *h*, however, an unlimited number of possible values could be elucidated by tuning *w*. Hence, the *h*‖*w*‖ was defined as one and try to minimize ‖*w*‖ by using following solution [40]:

$$ \min \frac{1}{2}{\left\Vert w\right\Vert}^2\; subject\kern0.17em to\;{c}^t\left({w}^T{x}^t+{w}_0\right)\ge +1,\forall t $$

In this work, SVM could be adopted to determine a hyperplane for discriminating between positive and negative instances with maximal margin in a vector space containing n dimensions (size of attribute set). The mass-to-charge ratio values of spectra were represented as a numeric vector in an n-dimensional vector space, which are the input values for SVM. A famous SVM public resource, called LIBSVM [41], was downloaded and installed in our computing server for an iterative training of multiple SVMs in accordance with various feature sets. In the machine learning problem, it has been demonstrated that if the best discriminant is nonlinear, instead of enabling a nonlinear modeling, we could map all n-dimensional vectors to new vector space with higher dimension m, where m > n, based on using nonlinear kernel functions. As demonstrated in previous methods [42–45], the radial basis function (RBF) was typically chosen as the specified kernel function on learning of SVM models. The RBF function was given as follows:

$$ K\left({x}^t,x\right)=\exp \left\{-\frac{{\left\Vert {x}^t-x\right\Vert}^2}{2{s}^2}\right\} $$

where *x*^*t*^ is the center and *s* is the radius, which should be provided by programmer. When using LIBSVM, cost (*c*) and gamma (*r*) are two supporting parameters used to optimize the radius of kernel function and softness of hyperplane, respectively. To achieve the feasible values of gamma (*r*) and cost (*c*) in model learning, an optimization program, written in Python, was provided by LIBSVM.

### Random forest

Random forest (RF) is a sort of ensemble model that involves the aggregation of multiple decision tree classifiers. Based on the integration of multiple decision trees within a RF model, each tree was generated from a subset of *k* attributes randomly selecting from training dataset with a total of *m* attributes, where *k* is less than *m*. In this way, we can obtain multiple decision-making results. Typically, the majority voting method is adopted to integrate the results to make a final decision, based on the class label with the most votes. The performance of a RF decision-making system is associated with the dimension of random vector, which is the number of attributes (*k*) used in each decision tree. The value of *k* is typically defined as

$$ k={\log}_2m+1 $$

where m is the total number of attributes in training dataset [46]. In this study, a package of random forest, which has been integrated into Weka toolkit [47], was utilized to construct RF classifiers based on various attribute sets.

### Five-fold cross validation and performance evaluation

Model training was built based on the input training data set, and two ML algorithms (i.e., SVM and RF) were used to generate the predictive models. Cross-validation was used to access both the variability of a data set and the reliability of any model trained using that data [48]. Cross-validation randomly splits the training data set into a number of partitions or folds. The module begins with setting aside the data in one part of folds for validation, and uses the remaining folds to train a model. To choose the best final model of each serotype, five-fold cross-validation was carried out for each of the different feature combinations to evaluate the predictive performances. The training data set was divided into five approximately equal sized subgroups. The ratio of the testing set and training set was 1:4 and the cross-validation process was repeated five times. The five validation results were combined to generate a single estimation.

To improve the reliability of performance, we used independent testing data set to test the optimal models with the best performance in cross-validation evaluation. The following measures were used for evaluating the performance of the predictive models, including sensitivity (Sn), specificity (Sp), accuracy (Acc), and Matthews correlation coefficient (MCC):

2 $$ {S}_n=\frac{TP}{TP+ FN} $$

3 $$ {S}_p=\frac{TN}{TN+ FP} $$

4 $$ Acc=\frac{TP+ TN}{TP+ TN+ FP+ FN} $$

5 $$ MCC=\frac{\left( TP\ast TN\right)-\left( FN\ast FP\right)}{\sqrt{\left( TP+ FN\right)\ast \left( TN+ FP\right)\ast \left( TP+ FP\right)\ast \left( TN+ FN\right)}} $$

where TP, TN, FP, and FN represented the number of true positives, true negatives, false positives, and false negatives, respectively.

## Supplementary information

**Additional file 1 Table S1.** The information of the relative works. MLST: multilocus sequencing typing; CC: clonal cluster. **Table S2.** The distribution of MLS types among various serotypes of GBS. Table **S3.** Most discriminative peaks (top 10) of each serotypes. The peaks were selected by OneR and sorted according to the importance score generated from OneR. **Table S4.** Most discriminative peaks (top 10) of each serotypes. The peaks were selected by PCC and sorted according to the importance score generated from PCC. **Table S5.** Data distribution of selected peaks between type Ia and non-type Ia. **Table S6.** Data distribution of selected peaks between type Ib and non-type Ib. **Table S7.** Data distribution of selected peaks between type III and non-type III. **Table S8.** Data distribution of selected peaks between type V and non-type V. **Table S9.** Data distribution of selected peaks between type VI and non-type VI. **Table S10.** Number of peak pairs for each serotype under various bin size. The peak pairs were selected by either OneR or PCC. **Figure S1.** Data distribution of training data set by pseudo gel views. **Figure S2.** Performance of machine learning models under different number of features, which were selected and ranked by OneR. **Figure S3.** Performance of machine learning models under different number of features, which were selected and ranked by PCC. **Figure S4.** The ROC curve of comparison the predictive models for each serotype when using OneR for feature selection with four-kind fold of cross validation (5, 10, 20, and 30-fold cross validation). **Figure S5.** The ROC curve of comparison the predictive models for each serotype when using PCC for feature selection with four-kind fold of cross validation (5, 10, 20, and 30-fold cross validation).

## Data Availability

The datasets used and analyzed during the current study are available from the corresponding authors on reasonable request.

## Abbreviations

Acc: Accuracy
CPSs: Capsular polysaccharides
DT: Decision tree
FN: False negative
FP: False positive
GBS: Group B streptococcus
LA: Latex agglutination
m/z: Mass-to-charge ratio
MALDI-TOF MS: Matrix-assisted laser desorption ionization time-of-fly mass spectrometry
MCC: Matthews correlation coefficient
ML: Machine learning
MLST: Multilocus sequence typing
OneR: One Rule
PCC: Pearson correlation coefficient
RF: Random forest
*S. agalactiae*: *Streptococcus agalactiae*
Sn: Sensitivity
Sp: Specificity
SVM: Support vector machine
TIC: Total ion count
TN: True negative
TP: True positive
UHCA: Unsupervised hierarchical cluster analysis
