# Supplementary material for: Rapid classification of group B Streptococcus serotypes based on matrix-assisted laser desorption ionization-time of flight mass spectrometry and machine learning techniques
Source: BMC Bioinformatics. 2019 Dec 24;20(Suppl 19):703. doi: 10.1186/s12859-019-3282-7 (PMC6929280; doi:10.1186/s12859-019-3282-7)
Supplement: Supplementary file 1 — Additional file 1 Table S1. The information of the relative works. MLST: multilocus sequencing typing; CC: clonal cluster. Table S2. The distribution of MLS types among various serotypes of GBS. Table S3. Most discriminative peaks (top 10) of each serotypes. The peaks were selected by OneR and sorted according to the importance score generated from OneR. Table S4. Most discriminative peaks (top 10) of each serotypes. The peaks were selected by PCC and sorted according to the importance score generated from PCC. Table S5. Data distribution of selected peaks between type Ia and non-type Ia. Table S6. Data distribution of selected peaks between type Ib and non-type Ib. Table S7. Data distribution of selected peaks between type III and non-type III. Table S8. Data distribution of selected peaks between type V and non-type V. Table S9. Data distribution of selected peaks between type VI and non-type VI. Table S10. Number of peak pairs for each serotype under various bin size. The peak pairs were selected by either OneR or PCC. Figure S1. Data distribution of training data set by pseudo gel views. Figure S2. Performance of machine learning models under different number of features, which were selected and ranked by OneR. Figure S3. Performance of machine learning models under different number of features, which were selected and ranked by PCC. Figure S4. The ROC curve of comparison the predictive models for each serotype when using OneR for feature selection with four-kind fold of cross validation (5, 10, 20, and 30-fold cross validation). Figure S5. The ROC curve of comparison the predictive models for each serotype when using PCC for feature selection with four-kind fold of cross validation (5, 10, 20, and 30-fold cross validation). [file 12859_2019_3282_MOESM1_ESM.docx]

**Supplementary materials**

**Table S1. The information of the relative works. MLST: multilocus sequencing typing; CC: clonal cluster.**

| Reference | Species | Typing methods | Cases number |
| --- | --- | --- | --- |
| Marie-Frédérique Lartigue, et al | Group B streptococcus | MLST/serotype | 197 |
| Hsiao-Chuan Lin, et al | Group B streptococcus | MLST/serotype | 104 |

**Table S2. The distribution of MLS types among various serotypes of GBS.** MLST: multilocus sequence typing. GBS: group B streptococcus.

| **MLST** | **Serotype** | | | | | | | |
| --- | --- | --- | --- | --- | --- | --- | --- | --- |
|  | **Ia** | **Ib** | **II** | **III** | **V** | **VI** | **VII** | **Total** |
| 1 | 4 | 2 | 13 | 4 | 26 | 70 | 1 | 120 |
| 3 |  | 1 | 1 |  |  |  |  | 2 |
| 7 | 2 |  |  |  |  |  |  | 2 |
| 10 |  | 1 |  |  | 1 |  |  | 2 |
| 12 |  | 37 | 1 |  | 1 | 1 |  | 40 |
| 17 |  |  |  | 78 |  |  |  | 78 |
| 19 |  |  |  | 20 | 2 |  |  | 22 |
| 23 | 19 |  |  | 1 | 2 |  |  | 22 |
| 24 | 12 |  |  |  |  |  |  | 12 |
| 26 |  |  |  |  | 1 |  |  | 1 |
| 27 |  |  |  |  | 1 |  |  | 1 |
| 110 |  |  |  |  | 1 |  |  | 1 |
| 144 | 1 |  |  |  |  |  |  | 1 |
| 153 |  |  |  |  | 1 |  |  | 1 |
| 268 | 2 |  |  |  |  |  |  | 2 |
| 335 |  |  |  | 3 |  |  |  | 3 |
| 438 |  |  |  | 1 |  |  |  | 1 |
| 651 | 1 |  |  | 1 |  |  |  | 2 |
| 687 |  |  |  | 1 |  |  |  | 1 |
| 705 |  |  |  | 1 |  |  |  | 1 |
| 714 |  |  | 1 |  |  |  |  | 1 |
| 862 |  |  |  | 1 |  |  |  | 1 |
| 890 |  |  |  |  | 3 |  |  | 3 |
| 920 |  |  |  |  |  | 2 |  | 2 |
| 922 |  |  |  |  |  | 1 |  | 1 |
| 929 |  |  |  |  | 2 |  |  | 2 |
| Total | 41 | 41 | 16 | 111 | 41 | 74 | 1 | 325 |

**Table S3. Most discriminative peaks (top 10) of each serotypes. The peaks were selected by OneR and sorted according to the importance score generated from OneR.**

| Peak No. | Type Ia | | Type Ib | | Type III | | Type V | | Type VI | |
| --- | --- | --- | --- | --- | --- | --- | --- | --- | --- | --- |
|  | **m/z** | **Score** | **m/z** | **Score** | **m/z** | **Score** | **m/z** | **Score** | **m/z** | **Score** |
| 1 | 2978 | 76.7 | 3819 | 72.7 | 7620 | 83.1 | 15466 | 72.2 | 4509 | 84.5 |
| 2 | 3820 | 73.2 | 6741 | 71.3 | 7639 | 76.3 | 3466 | 72.2 | 6892 | 82.0 |
| 3 | 3193 | 72.0 | 6653 | 71.0 | 2957 | 73.2 | 3013 | 72.0 | 6250 | 80.2 |
| 4 | 15181 | 71.3 | 6770 | 70.8 | 6892 | 71.7 | 7735 | 72.0 | 9017 | 78.2 |
| 5 | 7581 | 71.3 | 6237 | 70.5 | 2242 | 70.2 | 6252 | 71.7 | 9044 | 73.4 |
| 6 | 7509 | 71.0 | 9099 | 70.5 | 3812 | 70.2 | 6930 | 71.7 | 4514 | 70.7 |
| 7 | 3983 | 71.0 | 8212 | 70.5 | 2035 | 70.2 | 7489 | 71.5 | 6883 | 67.7 |
| 8 | 4453 | 70.8 | 10957 | 70.5 | 15496 | 69.8 | 6908 | 71.3 | 15469 | 67.4 |
| 9 | 5744 | 70.5 | 13545 | 70.5 | 5201 | 68.9 | 5405 | 71.3 | 6247 | 67.4 |
| 10 | 5955 | 70.5 | 14135 | 70.5 | 3447 | 68.9 | 10151 | 71.3 | 15080 | 66.9 |

**Table S4. Most discriminative peaks (top 10) of each serotypes. The peaks were selected by PCC and sorted according to the importance score generated from PCC.**

| Peak No. | Type Ia | | Type Ib | | Type III | | Type V | | Type VI | |
| --- | --- | --- | --- | --- | --- | --- | --- | --- | --- | --- |
|  | **m/z** | **Score** | **m/z** | **Score** | **m/z** | **Score** | **m/z** | **Score** | **m/z** | **Score** |
| 1 | 2978 | 0.431 | 2224 | 0.307 | 7620 | 0.599 | 6892 | 0.342 | 6251 | 0.526 |
| 2 | 3820 | 0.375 | 2226 | 0.270 | 6893 | 0.506 | 6250 | 0.329 | 4510 | 0.518 |
| 3 | 7639 | 0.264 | 10155 | 0.253 | 6250 | 0.458 | 6928 | 0.292 | 6891 | 0.481 |
| 4 | 7620 | 0.263 | 6892 | 0.251 | 2957 | 0.361 | 6909 | 0.264 | 9031 | 0.472 |
| 5 | 6250 | 0.261 | 6250 | 0.245 | 3820 | 0.359 | 7620 | 0.263 | 9046 | 0.457 |
| 6 | 6892 | 0.255 | 3820 | 0.245 | 7642 | 0.329 | 3466 | 0.231 | 3447 | 0.454 |
| 7 | 3092 | 0.233 | 2085 | 0.233 | 2082 | 0.322 | 3012 | 0.228 | 4515 | 0.437 |
| 8 | 3193 | 0.229 | 7639 | 0.230 | 7638 | 0.300 | 3469 | 0.214 | 9018 | 0.422 |
| 9 | 7488 | 0.222 | 5911 | 0.226 | 3811 | 0.295 | 2991 | 0.209 | 6248 | 0.322 |
| 10 | 4509 | 0.211 | 2409 | 0.225 | 7751 | 0.295 | 3030 | 0.207 | 9012 | 0.316 |

**Table S5. Data distribution of selected peaks between type Ia and non-type Ia.**

| Peak | Number of data in Type Ia | % | Number of data in non-Type Ia | % | PCC ranking | PMID |
| --- | --- | --- | --- | --- | --- | --- |
| 2978 | 54 | 44% | 243 | 86% | 1 |  |
| 3820 | 72 | 59% | 58 | 20% | 2 |  |
| 7639 | 96 | 78% | 141 | 50% | 3 |  |
| 7620 | 0 | 0% | 56 | 20% | 4 |  |
| 6250 | 15 | 12% | 109 | 38% | 5 |  |
| 6892 | 99 | 80% | 152 | 54% | 6 |  |
| 3092 | 54 | 44% | 195 | 69% | 7 |  |
| 3193 | 9 | 7% | 0 | 0% | 8 |  |
| 7488 | 3 | 2% | 55 | 19% | 9 |  |
| 4509 | 15 | 12% | 92 | 32% | 10 |  |

**Table S6. Data distribution of selected peaks between type Ib and non-type Ib.**

| Peak | Number of data in Type Ib | % | Number of data in non-Type Ib | % | PCC ranking | PMID |
| --- | --- | --- | --- | --- | --- | --- |
| 2224 | 12 | 10% | 116 | 41% | 1 |  |
| 2226 | 72 | 59% | 85 | 30% | 2 |  |
| 10155 | 75 | 61% | 96 | 34% | 3 |  |
| 6892 | 69 | 56% | 84 | 30% | 4 | 29317173 |
| 6250 | 6 | 5% | 74 | 26% | 5 |  |
| 3820 | 51 | 41% | 52 | 18% | 6 |  |
| 2085 | 63 | 51% | 77 | 27% | 7 |  |
| 7639 | 60 | 49% | 72 | 25% | 8 |  |
| 5911 | 0 | 0% | 43 | 15% | 9 |  |
| 2409 | 63 | 51% | 79 | 28% | 10 |  |

**Table S7. Data distribution of selected peaks between type III and non-type III.**

| Peak | Number of data in Type III | % | Number of data in non-Type III | % | PCC ranking | PMID |
| --- | --- | --- | --- | --- | --- | --- |
| 7620 | 51 | 46% | 0 | 0% | 1 | 21663770  23372719 |
| 6893 | 98 | 88% | 75 | 35% | 2 | 29317173 |
| 6250 | 5 | 5% | 108 | 50% | 3 |  |
| 2957 | 33 | 30% | 9 | 4% | 4 |  |
| 3820 | 4 | 4% | 78 | 36% | 5 |  |
| 7642 | 4 | 4% | 70 | 33% | 6 |  |
| 2082 | 31 | 28% | 11 | 5% | 7 |  |

**Table S8. Data distribution of selected peaks between type V and non-type V.**

| Peak (m/z) | Number of data in Type V | % | Number of data in non-Type V | % | PCC ranking | PMID |
| --- | --- | --- | --- | --- | --- | --- |
| 6892 | 30 | 24% | 175 | 62% | 1 |  |
| 6250 | 81 | 66% | 87 | 31% | 2 | 21663770  23372719 |
| 6928 | 75 | 61% | 85 | 30% | 3 |  |
| 6909 | 15 | 12% | 2 | 1% | 4 |  |
| 7620 | 0 | 0% | 56 | 20% | 5 |  |
| 3466 | 57 | 46% | 66 | 23% | 6 |  |
| 3012 | 12 | 10% | 2 | 1% | 7 |  |
| 3469 | 63 | 51% | 208 | 73% | 8 |  |
| 2991 | 15 | 12% | 6 | 2% | 9 |  |
| 3030 | 9 | 7% | 1 | 0% | 10 |  |

**Table S9. Data distribution of selected peaks between type VI and non-type VI.**

| Peak | Number of data in Type VI | % | Number of data in non-Type VI | % | PCC ranking | PMID |
| --- | --- | --- | --- | --- | --- | --- |
| 6251 | 106 | 72% | 47 | 19% | 1 | 29317173 |
| 4510 | 120 | 81% | 69 | 27% | 2 |  |
| 6891 | 2 | 1% | 118 | 47% | 3 |  |
| 9031 | 10 | 7% | 135 | 54% | 4 |  |
| 9046 | 60 | 41% | 11 | 4% | 5 |  |
| 3447 | 2 | 1% | 109 | 43% | 6 |  |
| 4515 | 4 | 3% | 109 | 43% | 7 |  |
| 9018 | 92 | 62% | 51 | 20% | 8 |  |
| 6248 | 38 | 26% | 10 | 4% | 9 |  |
| 9012 | 32 | 22% | 6 | 2% | 10 |  |

**Table S10. Number of peak pairs for each serotype under various bin size. The peak pairs were selected by either OneR or PCC.**

| Serotypes | OneR | | PCC | |
| --- | --- | --- | --- | --- |
|  | **Bin size (m/z)** | **Number of peak pairs** | **Bin size (m/z)** | **Number of peak pairs** |
| Ia | 9 | 26 | 9 | 28 |
| Ib | 7 | 31 | 3 | 35 |
| III | 9 | 34 | 6 | 33 |
| V | 1 | 16 | 9 | 30 |
| VI | 9 | 40 | 7 | 45 |


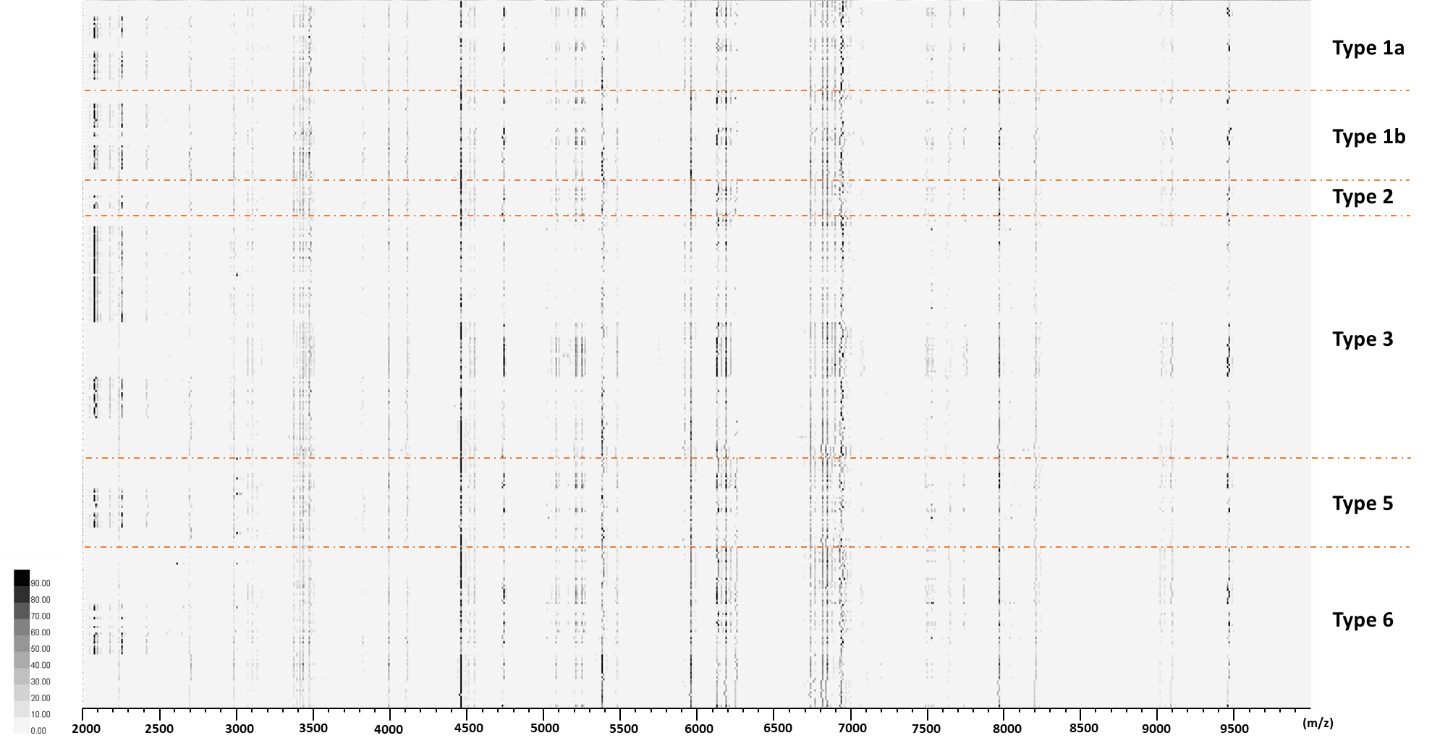


Figure S1. Data distribution of training data set by pseudo gel views.

| (a) 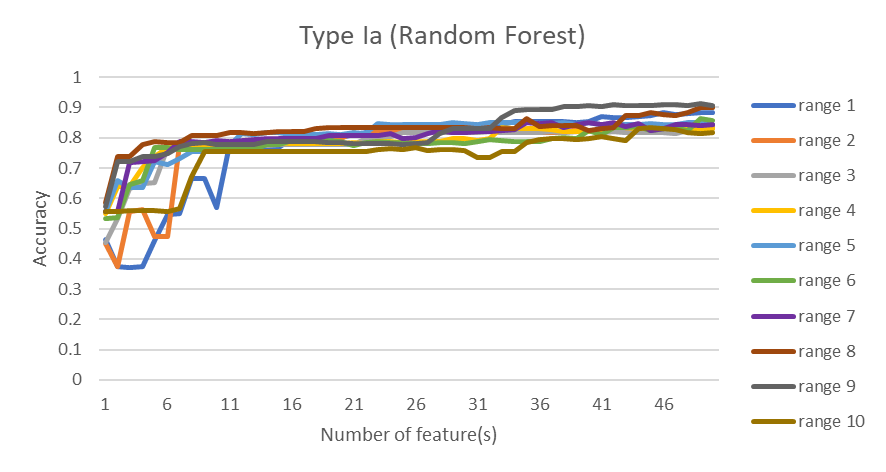 | (b)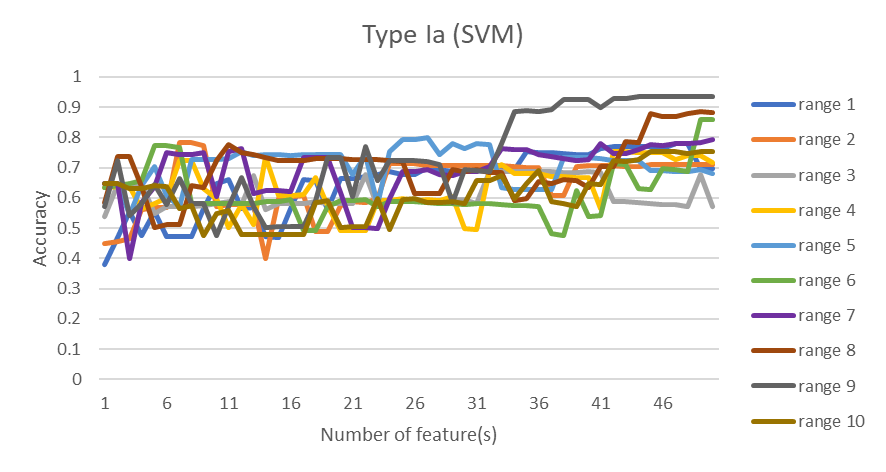 |
| --- | --- |
| (c)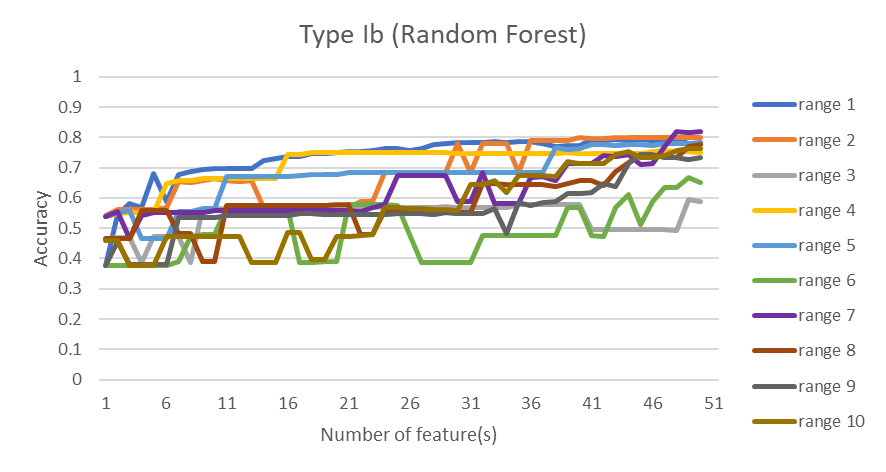 | (d)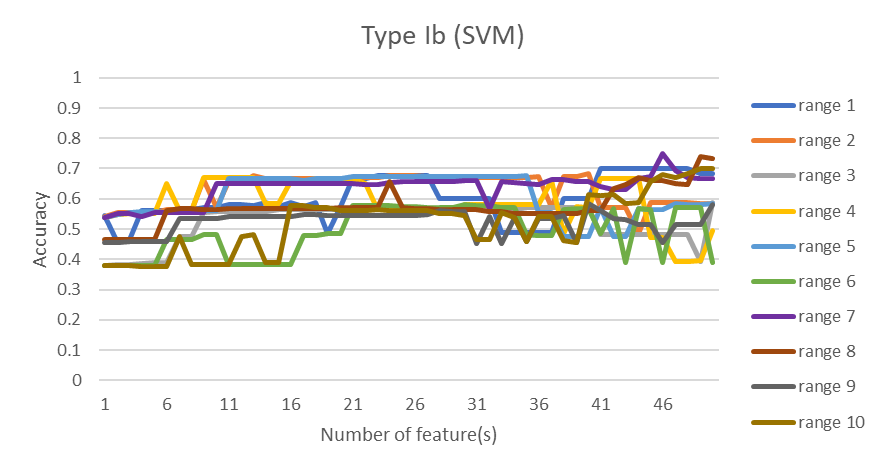 |
| (e)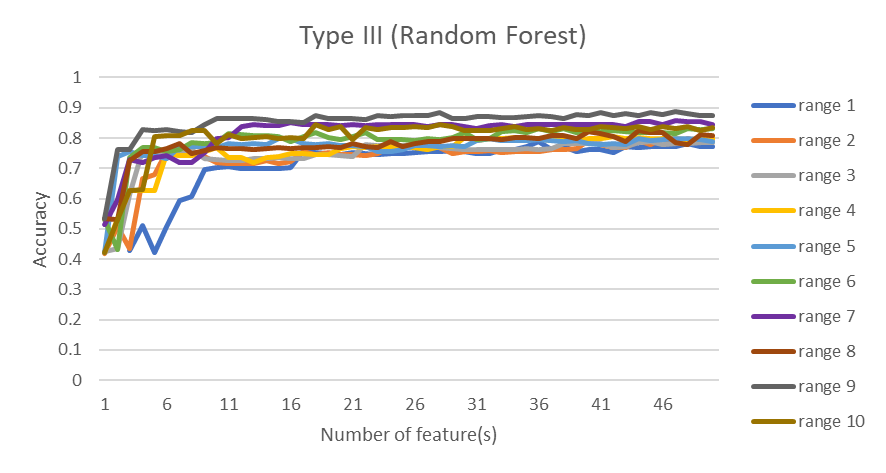 | (f)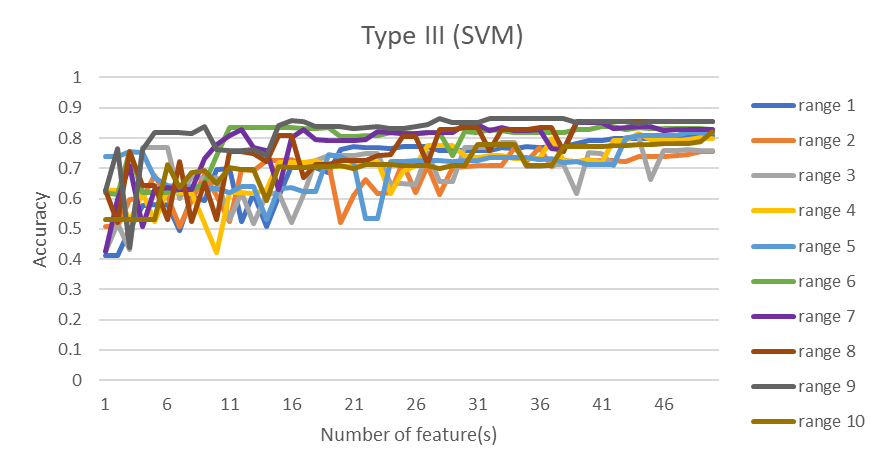 |
| (g)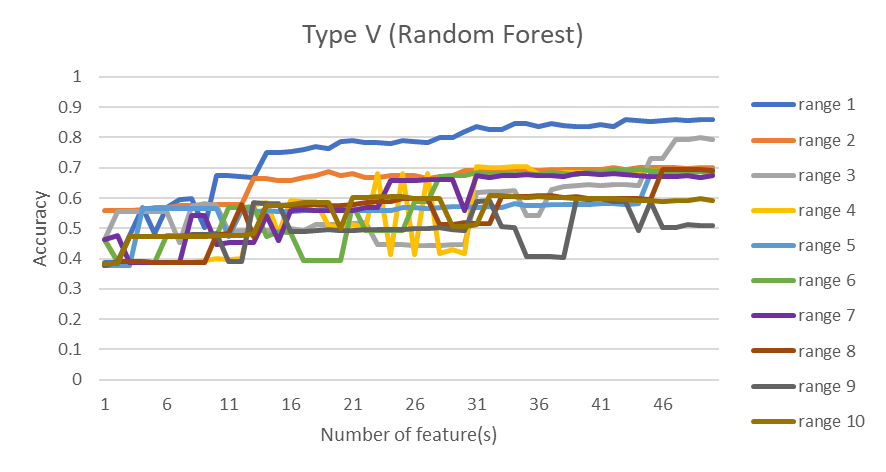 | (h)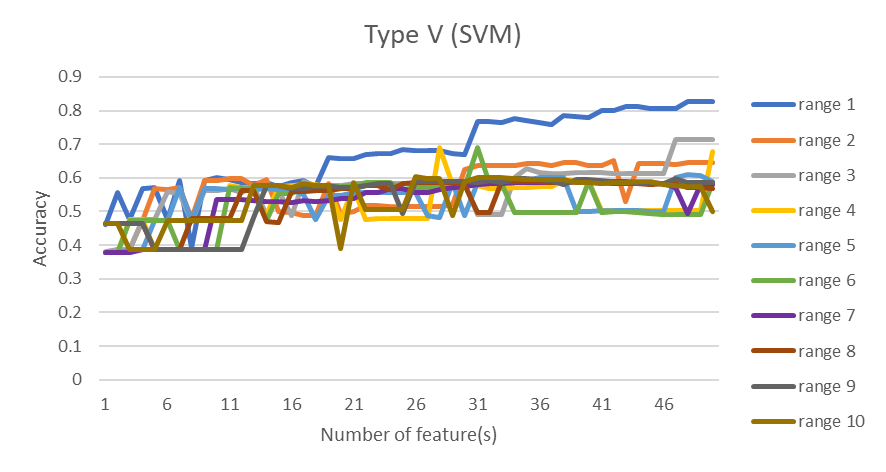 |
| (i)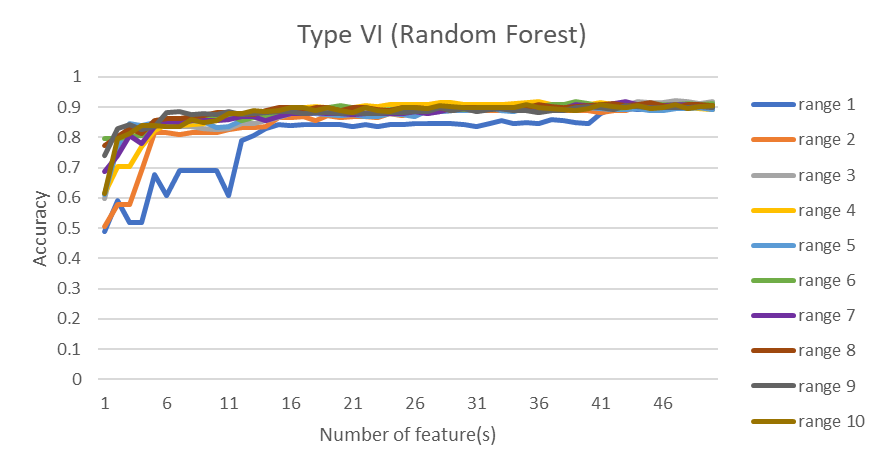 | (j)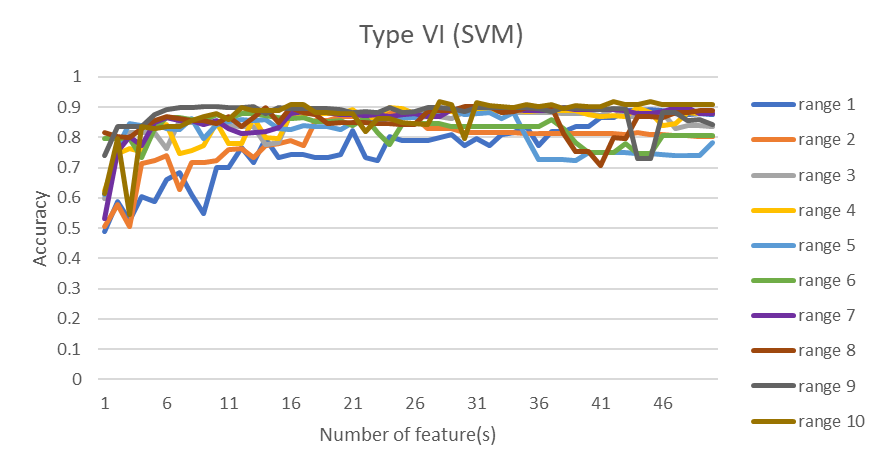 |

**Figure S2. Performance of machine learning models under different number of features, which were selected and ranked by OneR.** SVM: support vector machine.

| (a)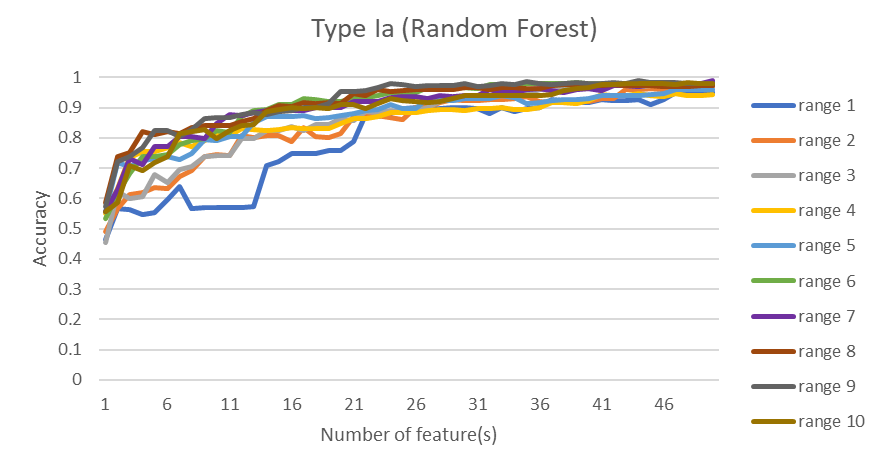 | (b)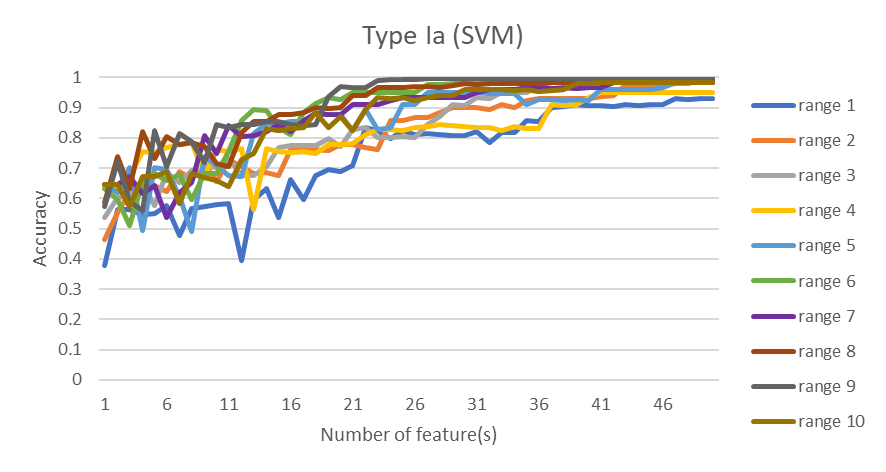 |
| --- | --- |
| (c)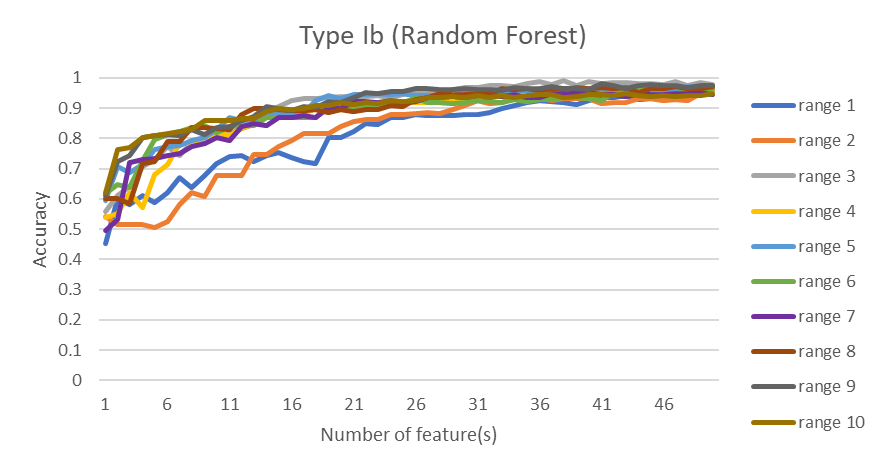 | (d)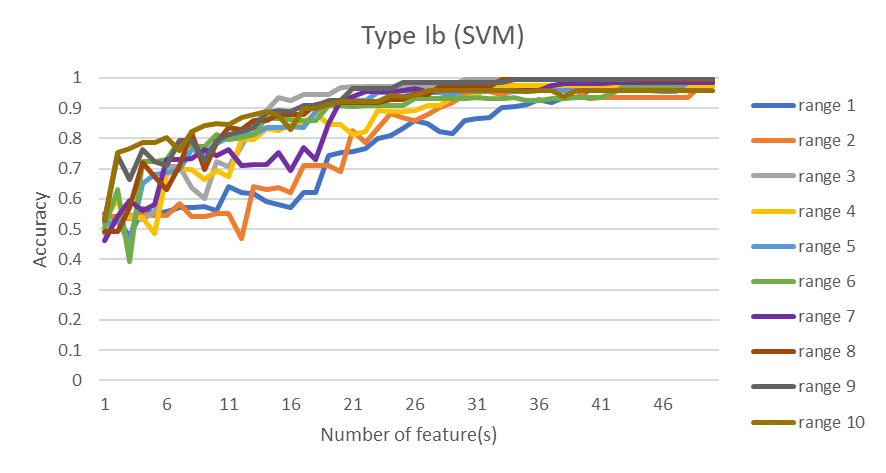 |
| (e)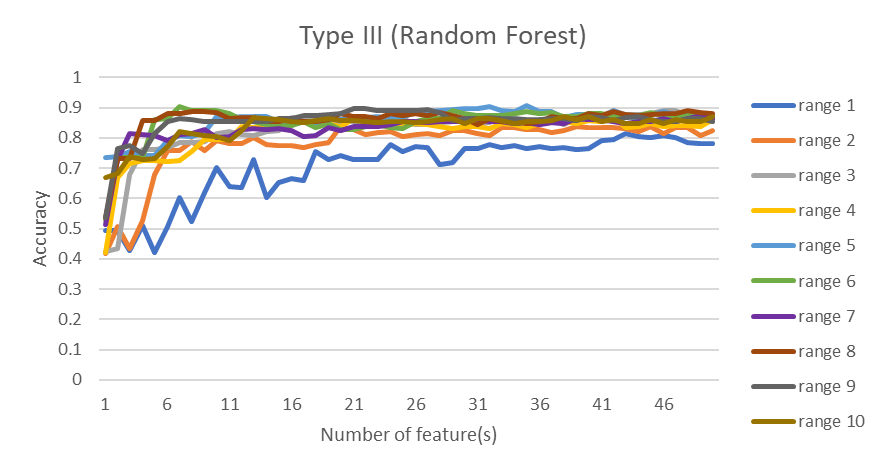 | (f)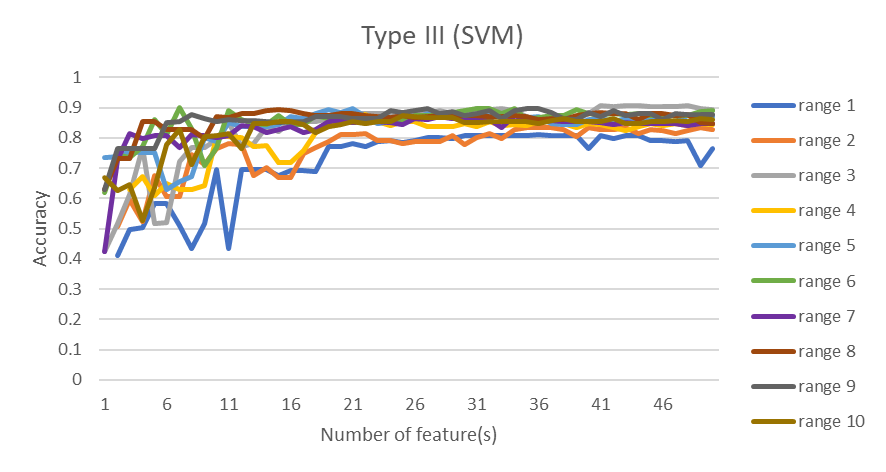 |
| (g)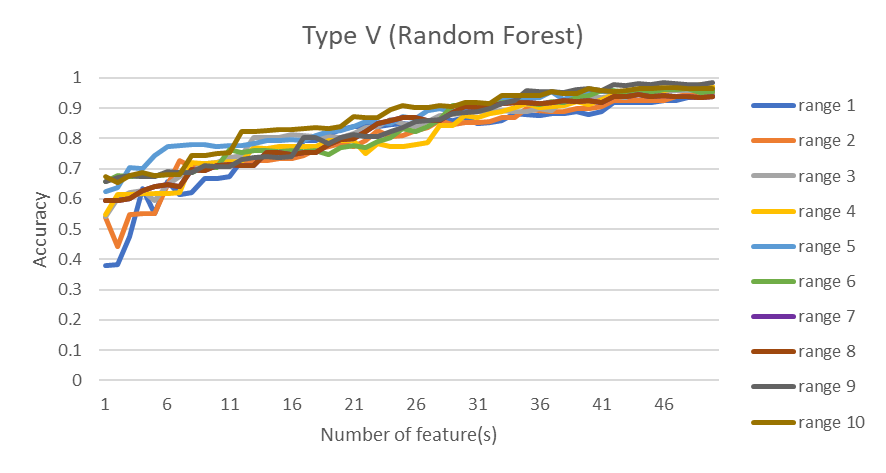 | (h)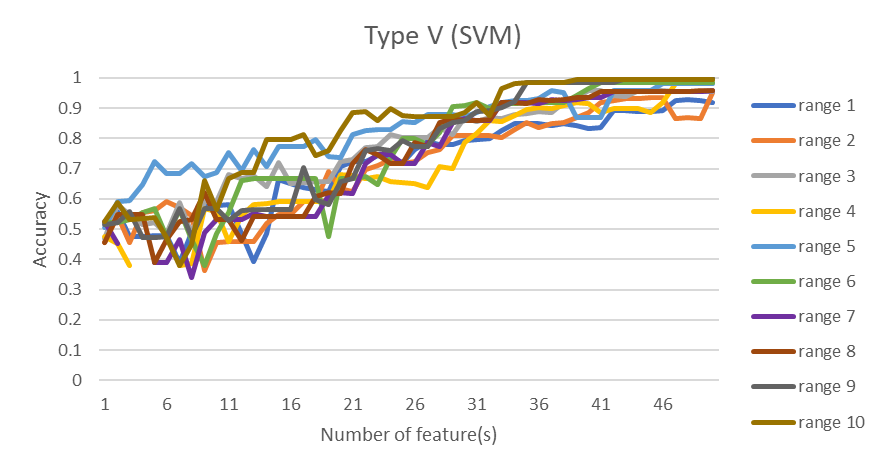 |
| (i)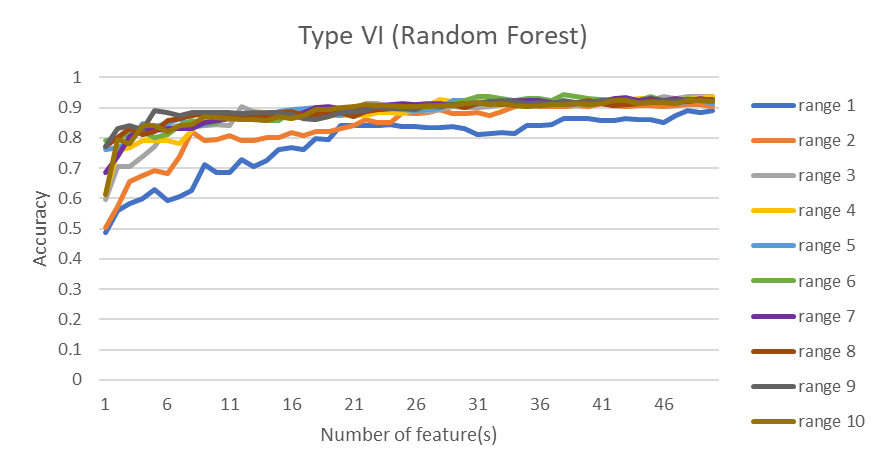 | (j)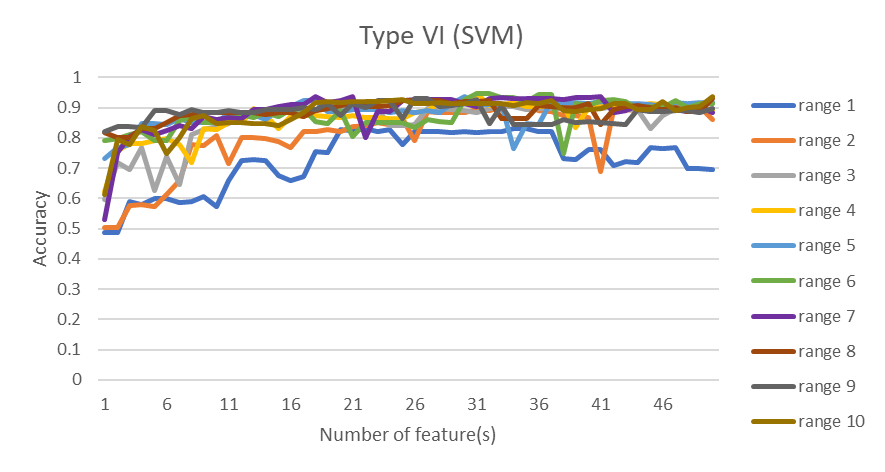 |

**Figure S3. Performance of machine learning models under different number of features, which were selected and ranked by PCC.** SVM: support vector machine.

| (a)Type Ia | (b)Type Ia |
| --- | --- |
| (c)Type Ib | (d) Type Ib |
| (e)Type III | (f)Type III |
| (g)Type V | (h)Type V |
| (i)Type VI | (j)TypeV |

**Figure S4. The ROC curve of comparison the predictive models for each serotype when using OneR for feature selection with four-kind fold of cross validation (5, 10, 20, and 30-fold cross validation).** SVM: support vector machine.

| (a)Type Ia | (b)Type Ia |
| --- | --- |
| (c) Type Ib | (d)Type Ib |
| (e) Type III | (f)Type III |
| (g) Type V | (h)Type V |
| (i) Type VI | (j)Type VI |

**Figure S5. The ROC curve of comparison the predictive models for each serotype when using PCC for feature selection with four-kind fold of cross validation (5, 10, 20, and 30-fold cross validation).** SVM: support vector machine.
